# Prediction of Permeate Flux in Ultrafiltration Processes: A Review of Modeling Approaches

**DOI:** 10.3390/membranes11050368

**Published:** 2021-05-18

**Authors:** Carolina Quezada, Humberto Estay, Alfredo Cassano, Elizabeth Troncoso, René Ruby-Figueroa

**Affiliations:** 1 Programa Institucional de Fomento a la Investigación, Desarrollo e Innovación (PIDi), Universidad Tecnológica Metropolitana, Santiago 8940577, Chile; carolina.quezadab@utem.cl; 2 Programa de Doctorado en Ciencia de Materiales e Ingeniería de Procesos (Doctoral Program in Materials Science and Process Engineering), Universidad Tecnológica Metropolitana, Santiago 8940577, Chile; 3 Advanced Mining Technology Center (AMTC), University of Chile, Av. Tupper 2007 (AMTC Building), Santiago 8370451, Chile; humberto.estay@amtc.cl; 4 Institute on Membrane Technology, ITM-CNR, via P. Bucci, 17/C, I-87030 Rende, Italy; a.cassano@itm.cnr.it

**Keywords:** ultrafiltration, phenomenological models, non-phenomenological models, permeate flux prediction

## Abstract

In any membrane filtration, the prediction of permeate flux is critical to calculate the membrane surface required, which is an essential parameter for scaling-up, equipment sizing, and cost determination. For this reason, several models based on phenomenological or theoretical derivation (such as gel-polarization, osmotic pressure, resistance-in-series, and fouling models) and non-phenomenological models have been developed and widely used to describe the limiting phenomena as well as to predict the permeate flux. In general, the development of models or their modifications is done for a particular synthetic model solution and membrane system that shows a good capacity of prediction. However, in more complex matrices, such as fruit juices, those models might not have the same performance. In this context, the present work shows a review of different phenomenological and non-phenomenological models for permeate flux prediction in UF, and a comparison, between selected models, of the permeate flux predictive capacity. Selected models were tested with data from our previous work reported for three fruit juices (bergamot, kiwi, and pomegranate) processed in a cross-flow system for 10 h. The validation of each selected model’s capacity of prediction was performed through a robust statistical examination, including a residual analysis. The results obtained, within the statistically validated models, showed that phenomenological models present a high variability of prediction (values of R-square in the range of 75.91–99.78%), Mean Absolute Percentage Error (MAPE) in the range of 3.14–51.69, and Root Mean Square Error (RMSE) in the range of 0.22–2.01 among the investigated juices. The non-phenomenological models showed a great capacity to predict permeate flux with R-squares higher than 97% and lower MAPE (0.25–2.03) and RMSE (3.74–28.91). Even though the estimated parameters have no physical meaning and do not shed light into the fundamental mechanistic principles that govern these processes, these results suggest that non-phenomenological models are a useful tool from a practical point of view to predict the permeate flux, under defined operating conditions, in membrane separation processes. However, the phenomenological models are still a proper tool for scaling-up and for an understanding the UF process.

## 1. Introduction

Membrane processes have become major techniques in the food industry over the last few decades, thanks to their ability to provide gentle treatment of products at low-to-moderate temperatures.

Membrane applications in the food industry have focused on separation, fractionation, purification, clarification, and concentration of several food products and by-products such as whey, milk, wine, beer, vinegar fruit, and vegetable juices [1]. Typical advantages over conventional separation systems include high separation precision, better selectivity, operation at room temperature, no chemical damage, high automation, easy operation, energy saving, reduced cost, comprehensive utilization of resources, and reduced pollution. For these reasons, membrane processes are often recognized as the best available technology (BAT) in the food industry [2,3,4]. Among pressure-driven membrane processes, ultrafiltration (UF) has been extensively applied in the treatment of industrial effluents [5,6,7,8,9,10], oil-based emulsions [11,12,13,14], biological macromolecules [15,16,17], milk [18,19,20], sugar cane [21,22], extracts of soybean flour [23], clay suspensions [24], black kraft liquor [25], and fruit juices [26,27,28,29,30,31,32,33,34,35,36,37] among others. Within the fruit juice industry, bergamot, kiwifruit, and pomegranate have great importance in the market, not only for their volume of production, but also because they are characterized by a high concentration of phytochemicals which are recognized to be associated with antioxidant activities within others. Bergamot (Citrus bergamia, Risso) is an evergreen tree almost exclusively grown on the Ionian and Tyrrhenian Coast of Reggio Calabria Province (South Italy) with a production of 18,750 tons in 2017 [38], representing a significant economic benefit. Bergamot has been mainly cultivated to extract essential oils with applications in food, cosmetic and pharmaceutical industries [39] because of their high content of phytochemicals such as flavanone glycosides, limonoids, and quaternary ammonium compounds, all health-beneficial biomolecules [40,41]. On the other hand, Bergamot juice is considered a residue for its bitter taste; however, this juice is characterized by a large quantity and variety of nutraceuticals such as naringin, neoeriocitrin, neohesperidin, rutin, neodesmin, rhoifolin, and poncirin with demonstrated health implications [26]. Kiwifruit is another fruit with a high content of phytonutrients, including carotenoids, lutein, phenolics, flavonoids, vitamin C, and chlorophyll, all of them with strong antioxidant activity [31,32,33,42]; therefore, it offers benefits for specific health conditions and, consequently, it has a great potential for industrial exploitation. Italy, as the major producer worldwide, has a production of 330,000 tons/year (corresponding to 33% of the world production) principally in the regions of Latium, Emilia-Romagna, Piedmont, and Apulia [32]. Pomegranate (*Punica granatum* L.) is located in many different geographical regions, including tropical and subtropical regions. The leading producer locations include Mediterranean countries, India, Iran, and California [43]. Since several authors reported the therapeutic benefits of its consumation including antioxidant, antimicrobial, anti-carcinogenic, and anti-inflammatory properties, increased interest has been garnered for this fruit [44]. Polyphenolic compounds, including ellagotanins, anthocyanins, ellagic acid, and minerals, potassium, magnesium, and copper, are associated with a beneficial effect on health. The global pomegranate market was valued at USD 8.2 billion in 2018 and is expected to reach USD 23.14 billion by 2026 at a Compound Annual Growth Rate (CAGR) of 14.0 percent. Its widespread popularity drives increasing demand for pomegranate and its derivatives (such as pomegranate powder, pomegranate juice, functional beverages) as well as other pomegranate-derived products as a functional food and a source of nutraceuticals [43].

Regarding membrane processes, permeate flux in UF is one of the most critical parameters for evaluating membrane performance. Indeed, the evaluation of permeate flux, usually expressed as volume (or weight) per unit membrane area per unit time, is a critical issue in the projection of scaling-up from experience at the laboratory scale to pilot or industrial plants for a specific volume treatment requirement [45]. Thus, it is a crucial task to forecast permeate flux in long-term operations.

In this context, it is known that permeate flux is affected by several factors, including feed characteristics, membrane materials and properties, and operating conditions as well [46]. The reduction in membrane flux below that of the corresponding pure solvent flow over time promoted by membrane fouling leads to losses in productivity and higher operating costs as a result of higher energy cost, and maintenance [47,48]. In some cases, severe permeate flux reductions have been one of the main drawbacks for wide applications on an industrial scale [46,49,50,51].

Membrane lifetime and permeate flux are affected by the phenomena named concentration polarization (i.e., solute build-up) and fouling (e.g., microbial adhesion, gel layer formation, and solute adhesion) at the membrane surface [52,53]. Concentration polarization (CP), produced by the accumulation of soluts on the membrane surface, causes an increased resistance to solvent transport and possibly a change in the separation characteristics of the membrane. CP is considered as a reversible phenomena and is the primary reason of flux decline during the initial period of operation. Even though CP is considered reversible, it can lead to irreversible phenomena such as solute adsorption, solute precipitation, and gel layer formation as well [54]. On the other hand, irreversible fouling is caused by adsorption and obstruction phenomena inside the membrane pores [52]. The understanding of membrane fouling allows the limit or avoid its generation or reduce its effects by an adequate selection of membrane characteristics and the system’s operational conditions [55].

Transport mechanisms in the UF process are usually described by phenomenological models [56]. In the literature, more than 2840 articles have been published on the modeling of UF systems in the 1961–2019 period (data obtained in Google Scholar in July 2020 with the following keywords: ultrafiltration membrane, permeate flux, and modeling). This considerable number of articles includes phenomenological and non-phenomenological models, as well as the development of new models or modification of the traditional ones applied for a particular matrix. Despite the high quantity of articles related to modeling in UF until now, there is not an available review that summarizes all of them, or the most relevant ones. Moreover, it is necessary to address not only the description of the different models, but also to analyze and validate the capacity of permeate the flux prediction of selected models. This is done with the purpose of developing new models in which more realistic assumptions can be incorporated in phenomenological models for complex matrices such as fruit juices. This would improve the prediction capacity which will directly impact the use of these models for the scaling-up of processes from laboratory to pilot or industrial dimensions.

Published models to predict the permeate flux decline can be grouped into phenomenological [57,58], empirical [59], and semi-empirical [60] models. In the majority of cases, these models are based on convective transport under a pressure gradient and separation by size exclusion [61], diffusive transport through the cake layer [62,63,64], and fouling mechanisms [60,65,66,67] as well. Other models, unrelated to the phenomenological aspects, are based on statistical tools such as artificial neural networks, data mining, computational models of system dynamics, and principal component analysis (PCA) [8,12,15,68,69].

Even though there are some phenomenological [6,9,11,68,70,71] and non-phenomenological models [12,15,72,73] with applications at the pilot scale, the majority of developed models have been tested with ideal matrices (e.g., PEG, BSA, Dextran), and their predictions have been validated at the laboratory scale for short-term operations [69]. In this regard, Chew et al. [74] mentioned that, commonly, rigorous pilot-scale studies are not usually performed in the industrial practice due to the urgency of production and insufficient allocation for pilot studies. Thus, the natural question for people working in the field of membrane technology is related to the efficiency of these models in terms of permeate flux prediction, with more complex matrices such as fruit juices, dairy products, and by-products, oil derived effluents, and wastewaters in long-term operations.

In this context, this work aims to provide an extensive review illustrating the models considered as the foundation of the analysis of phenomenology in membrane separation (e.g., Carman–Kozeny equation, film theory, Darcy law) and relate them to how modeling continuously became more accurate in order to improve the capacity of explaining the complexity of membrane separation. This work reviews a series of models developed for permeate flux prediction in UF, including phenomenological models (concentration polarization, osmotic pressure, resistance in series models) and non-phenomenological models. In addition, the advantages and disadvantages of these models are analyzed and discussed. Finally, an analysis of the capacity to predict permeate flux was developed for selected models (based on the criteria of a number of citations and validation within others) and tested for data related to the clarification of bergamot, kiwi, and pomegranate juices with UF membranes in long-term operations, as reported in previous studies [69].

## 2. Theory

Regarding filtration, Carman [75,76] was the first one to propose a relationship for an aggregate cake, where the prediction of permeate flux is related to the structure parameters of the cake layer, including mean particle size and thickness [77]. This relationship is known as the Carman–Kozeny equation.

In a pressure-driven filtration process such as UF, the pure solvent flux (commonly water) through a porous membrane is directly proportional to the applied hydrostatic pressure, according to:

(1) $$J_{w}=L_{p}\Delta P=\frac{\Delta P}{\mu_{w}R_{m}}$$

where *J_w_* is the solvent permeate flux, Δ*P* is the transmembrane pressure, *L_p_* is the membrane permeability, *µ_w_* is the solvent viscosity, and *R_m_* is the intrinsic membrane resistance. However, when solutes are added to the solvent, the behavior is entirely different [78]. This means that the flux would increase up to a certain limit. In this regard, Field et al. [65] introduced the concept of critical flux for microfiltration, stating that there is a permeate flux below which fouling is not observed. For operational curves of permeate flux versus transmembrane pressure, three areas or zones related with membrane fouling were described: a subcritical zone (Zone 1), where the transmembrane pressure is low, in which only the concentration polarization phenomenon exists and the permeate flux is lower than the critical flux; a Zone 2, characterized by the formation and consolidation of the cake layer, where pore blocking or particle adsorption can also occur; a Zone 3 due to the compaction of the cake, which is undesirable because it represents irreversible fouling, which is difficult to remove even using chemical membrane cleaning. The critical point, or critical transmembrane pressure, and the limiting point, which is the maximum permeate flux where the increase of permeate flux is not possible after a certain point, can be distinguished in the critical flux theory [18]. The limiting flux is affected by shear stress applied to the system as well as by the feed and module characteristics.

**Figure 1:**
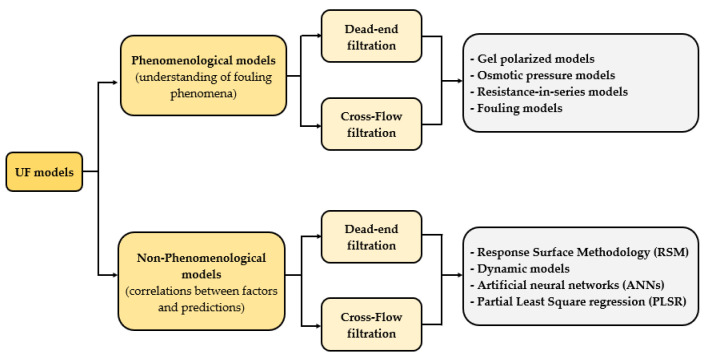
Classification of models developed for MF and UF processes.

Despite the importance of the critical flux theory as an operational parameter, the majority of the models developed for UF addressed in this review are focused on the prediction of permeate flux over operating time, but they are not focused on the determination of the limiting point or maximum permeate flux. In this regard, from the Carman-Kozeny equation until these days, several models have been developed as a tool to both describe the reduction in flux and to understand different phenomena involved in membrane filtration, since the understanding of how these factors affect membrane performance is crucial for equipment design [79,80]. Ohanessian et al. [81] mentioned that membrane models available in the literature could be classified into two categories: the end-use, such as permeate flux prediction, and the understanding of the fouling phenomenon. Some authors [1,80,82,83,84] have said that the models applied in UF for flux prediction can be grouped into five categories: (i) concentration polarization models; (ii) osmotic pressure models; (iii) resistance-in-series models; (iv) fouling models, based on the classical film theory model; and (v) non-phenomenological models. Figure 1 summarizes these categories, including models used to predict permeate flux in both MF and UF processes.

### 2.1. Concentration Polarization Models

This category includes all the models used to predict permeate flux, in which the concept of concentration polarization is the core of the model structure. In particular, it should be mentioned that film theory, applied to describe the mass transfer in systems in which fluid phases are present, was the first model to consider a resistance, such as concentration polarization, to the mass transfer, as shown in Equation (2):

(2) $$J=k\left(\frac{C_{g}-C_{p}}{C_{b}-C_{p}}\right);\;k=\frac{D}{\delta }$$

where *J* is the flux through the membrane, *C_p_* is the permeate concentration, *C_b_* is the bulk stream concentration, *C_g_* is the gel concentration at the membrane surface, *D* is the diffusivity coefficient, *δ* is the boundary layer thickness, and k is the mass transfer coefficient. This relationship includes phenomena occurring in UF processes, where solutes as macromolecules or colloids are conveyed by permeate flux to the membrane surface, and a portion of them is rejected by the membrane and diffused back into the bulk. In this regard, Aimar and Sanchez (1986) [85] have shown that the subsequent decrease in mass transfer coefficient can explain a limiting flux. They used the theories developed and quantified for heat transfer to membrane processes. In particular, the heat transfer work on transfer coefficient variations, theoretically established by Field (1990) [86], was combined with a mass transfer film theory in order to examine the limiting-flux phenomenon. In this context, the rejected solutes tend to form a gel layer on the membrane surface, which acts as an additional resistance [83]. This model assumes that *C_g_* is constant, and the flux of solvent is dependent only on the characteristics of *D*, *C_g_*, and *δ*. Fane et al. [87] have applied a correction based on the effective free area correction modifying the assumption in the conventional model for concentration polarization that implies a homogeneously permeable membrane surface. These authors described the membrane surface as a mosaic of regions of different solvent permeabilities depending on the manufacturing process and the structural changes caused by usage, damage to the membrane surface, and plugging of pores, among others. Blatt et al. [2], said that the hydraulic permeability of a gel or concentrated dispersion of submicroscopic particles is a complex function of the solid’s concentration and such variables as the size, shape, resistance, and state of aggregation of particles or molecules comprising the solid phase. In turn, Jonsson [88] indicated that even though polarization phenomena at the membrane–solute interface are usually characterized by the film-theory where the longitudinal mass transport within the boundary layer is assumed negligible, the effect of pressure impacts the permeate flux. This author established that it had been observed that as pressure is increased, permeate flux first increases and then remains more or less pressure independent (phenomena first explained by Blatt et al. [2]). It should be pointed out that the effect of Δ*P* was not considered by film theory; therefore, models including it can improve the capacity of prediction. Bacchin et al. [24] proposed a model which combined a contribution of both cake filtration and deposition kinetics on fouling. In this regard, the cake filtration law describes the fouling resistance as the sum of the membrane hydraulic resistance (*R_m_*) and cake resistance (R_c_). The latter resistance is assumed proportional to the amount deposited on the membrane (M_d_) and the cake-specific resistance (α). The deposition rate to the interface is expressed as the amount brought by convection (J_c_), minus a back flux (n). Considering the complexity of phenomena involved in membrane filtration, as well as the drawbacks reported in the literature for film theory, a series of modifications to the original model have been developed to date. Table 1 depicts a compilation of the classical and most-used models (considering the number of citations and validations), including the concentration polarization phenomena in the equation.

In order to overcome the drawbacks associated with film theory, several authors started to analyze and develop new models of concentration polarization phenomena. Michaels [98] and Blatt et al. [2] presented models for the concentration polarization of macrosolutes and colloids, in which the back-transport rate of concentrated solute controls the permeate flux. On the other hand, Porter [99] reported that the mass transfer from the membrane surface into the bulk stream is influenced by some forces other than the concentration gradient. This author described the so-called tubular pinch effect (effect appreciated in many colloidal suspensions in which a lesser frictional pressure drop would be expected from the fluid viscosity) is responsible for the increase in the mass transfer [99]. In this regard, other authors have also reported that film theory is not suitable for permeate flux prediction. Shen and Probstein [100] and Probstein et al. [101,102] reported modifications to film theory, including some transport properties such as viscosity and diffusion coefficient, in order to improve its prediction capacity. In particular, these authors successfully evaluated the diffusion coefficient with the gel layer in steady-state using parallel plate laminar UF [62].

Furthermore, Zydney and Colton [63] proposed a modification through the addition of the shear-enhanced diffusivity of large particles, which arises from mutually induced velocity fields in the shear flow of the concentrated suspension. The model was validated using a complex matrix such as blood solution in a cross-flow system with a tubular membrane. In comparison, Blatt [2] and Shen and Probstein [100] worked with a solution of BSA under ideal conditions where the models showed good predictions [103,104].

Although the film theory is the basis for understanding mass transport in a membrane, a series of modifications have been made to increase the prediction capacity [62]. Trettin and Doshi developed a model based on film theory in which the diffusivity of the system was included [62,83,105]. This model assumes that a gel concentration is reached instantaneously, and the diffusion coefficient is constant. The authors compared their model performance with film theory in a dead-end system with a stirred cell module using a BSA model solution. As expected, they found that film theory had a lower capacity of prediction in comparison to their model. Subsequently, Davis and Leighton [106] showed a model that described particle transport when there is a concentration polarization on the membrane surface under laminar flow. They established that the shear-induced concept (i.e., associated with a diffusion mechanism) could describe the lateral migration of particles from the porous wall [57,106,107]. In this regard, Romero and Davis [107] explained that the concentration profile could be determined by a differential mass balance, including convective transport near the membrane surface, whereas the diffusion transport is considered in bulk. In 1992, Davis [57] mentioned that the steady-state cake thickness and permeate flux are governed by the concentration polarization layer adjacent to the cake of rejected particles on the membrane surface. Depending on the characteristic particle size and the tangential shear rate, Brownian diffusion, shear-induced diffusion, or inertial lift can be considered the dominant mechanism for particle back-transport in the polarization layer. For typical shear rates, Brownian diffusion is important for submicron particles, the inertial lift is important for particles larger than approximately 10 microns, and shear-induced diffusion is dominant for intermediate-sized particles. In this regard, Davis [57] has simplified the previously published model [107], developing a shear-induced diffusion model, which can be used for permeate flux prediction based on the gel layer concept. On the one hand, Song and Elimelec [90] developed a model based on the concentration polarization phenomena for non-interacting particles in crossflow filtration. According to this model, a polarization layer exists directly over the membrane surface when the dimensionless filtration number defined in the model is lower than a critical value. In these conditions, pressure and temperature determine the wall particle concentration. On the other hand, when the filtration number is higher than the critical value, a gel layer of retained particles is formed between the polarization and membrane surfaces. The grade of polarization could be thus easily determined using this model. After that, Song (1998) [108] developed a model in which fouling is perceived as a dynamic process from a non-equilibrium stage to an equilibrium one. Under the influence of the boundary condition, equilibrium is firstly reached at the initial section of the crossflow filter, and the front of the equilibrium region progresses with time towards the end of the filter [108]. Other authors tested this model with PEG [45,55,109] and silica colloids (P50 and P0L) [110], highlighting a low capacity of prediction at the beginning of the process. Consequently, Singh et al. [64] analyzed the permeate flux in the clarification of synthetic fruit juice with a spiral-wound UF membrane module on the basis of Brownian diffusion, shear-induced, and combined diffusion models. They observed that the Brownian diffusion model was the best to predict experimental data.

All the models mentioned until now, based on concentration polarization, have a series of assumptions that limit the prediction capacity in complex matrices, such as those containing different components as in fruit juices. Some of these models base their performance on hydrodynamic diffusion, assuming that the tangential flow compensates for the convective transport on the membrane surface; therefore, the gel layer growth is controlled until it reaches a constant value [70]. The mathematical structure of these models is based on the quantification of the stationary gel layer [57]. However, this assumption has been questioned because the gel layer is variable during filtration [61,111]. Accordingly, another questioned assumption is the period in which the gel layer is formed. Some models assume that the formation is instantaneous; therefore, flux decay must be attributed only to this effect.

There are few recently published models in which concentration polarization is included. New theories have been developed thanks to improvements in the modeling field, since they consider the drawbacks mentioned inside the series of assumptions considered in this kind of model.

### 2.2. Osmotic Pressure Models

In cases where the solute concentration at the membrane surface is higher than the bulk concentration, the osmotic pressure of the feed solution at the membrane surface cannot be negligible. At this point, any increase in the pressure is partly canceled by the osmotic pressure increase. Osmotic pressure at a high concentration of solutes sharply increases due to strong solute–solute interactions [50]. The quantification of osmotic pressure for many macromolecular (polymers) solutions can be expressed in the form of a virial expansion:

(3) $$\pi =B_{1}C_{p}+B_{2}C_{p}^{2}+B_{3}C_{p}^{3}$$

where *B*_1_, *B*_2_, and *B*_3_ are the osmotic virial coefficients, and *C_p_* is the concentration of the macromolecular solution (g L^−1^). The *B*_1_ coefficient describes the so-called van’t Hoff’s limiting law for osmotic pressure, which is applicable at very dilute concentrations [50]. The osmotic pressure models consider that the flux is limited by the high osmotic pressure arising in the concentration-polarized layer in the membrane interface. Once the gel layer is formed on the membrane surface, the osmotic pressure plays a key role in permeate flux decay [83]. In this regard, Kedem and Katchalsky [112] were the first authors to develop a model including the osmotic pressure for the permeate flux prediction (shown in Equation (4)).

(4) $$J=\frac{\left|\Delta P\right|-\left|\Delta \pi \right|}{\mu R_{m}}$$

where ∆*π* is the osmotic pressure difference, ∆*P* is the transmembrane pressure, *µ* is the viscosity, and *R_m_* is the membrane resistance. From the model developed by Kedem and Katchalsky [112], several publications and models have been developed in which osmotic pressure is integrated directly in the equation or is related to other parameters included in the model. Table 2 summarizes the models with major number of citations and validated, which includes, directly or indirectly, osmotic pressure.

Goldsmith [118] mentioned that based on molecular weight consideration, the osmotic pressure of macromolecular solutions would appear to be insignificant. However, this author showed that polymer solutions such as dextran and polyethylene glycol fractions with concentrations over 1% *w*/*w* showed osmotic pressure exceeding 10 to 50 psi. Other authors mentioned that in cases where a consolidated gel layer exists, the rejection of low molecular weight molecules is observed, and, therefore, osmotic pressure cannot be negligible [83,118,124]. This behavior can be appreciated in clarifying fruit juices, where monosaccharides, like glucose and fructose, are present. In this regard, Wijmans et al. [111] determined that osmotic pressure limitation is more likely than gel layer limitation in molecular weight solutes in the range of 10–100 kDa. These authors remark that the osmotic pressure model does not predict a fully limiting flux, and contrary to the gel layer model, the osmotic pressure model explains the deviation of the permeate flux from the pure solvent flux at low pressures. At high-pressure differences, the dependency of the permeate flux on the pressure difference decreases gradually [61,111]. It should be mentioned that it has been demonstrated that osmotic pressure has a significant effect on permeate flux decay [2,98]. However, this conclusion contradicts what is reported in film theory, where osmotic pressure is considered insignificant. Therefore, the inclusion of osmotic pressure in the predictive models of UF processes requires a good knowledge of the matrix to be processed, particularly the presence of low molecular weight components and the selected membrane. Wijmans et al. [111] suggested that there is a point in which ΔP does not produce a significant effect on permeate flux because the permeate flux is limited by the osmotic pressure, which exerts a contrary influence, decreasing it [111,119]. Bhattacharjee et al. [58] tested the effect of osmotic pressure in permeate flux drop using solutions of PEG in a UF system. In this work, the authors developed an integrated model, including osmotic pressure and gel layer. They demonstrated that the use of gel layer models previously developed [105] for the prediction of permeate flux in low molecular weight solutions is not the correct path, and osmotic pressure must be considered. In this regard, Bhattacharya et al. [21] also proposed a model for sugar filtration in a stirred-cell in which the prediction of permeate flux is based on two equations, which include both the steady and non-steady states. The authors highlighted the difficulty of predicting the permeate flux in a steady-state in which osmotic pressure is mainly responsible for the permeate flux drop. Alternatively, Sarkar et al. [122] developed a sophisticated model based on osmotic pressure and tested it in a stirred-cell in which osmotic pressure can be related to the solute concentration.

### 2.3. Resistance-in-Series Models

The resistance-in-series models are based on the prediction of the permeate flux as a function of different resistances affecting membrane filtration. All these models use the concept developed by the Darcy law. This category of models is similar to those of osmotic pressure in which other phenomena such as absorption, blocking pores, and gel formation are included [125,126].

Permeate flux is usually written in terms of Δ*P* and total resistance as follows:

(5) $$J=\frac{\Delta P-\Delta \pi }{\mu R_{t}}$$

*R_t_* is the total resistance given by:

(6) $$R_{t}=R_{m}+R_{cp}+R_{f}+R_{g}\;$$

where *R_m_* the membrane resistance, *R_cp_* the concentration polarization resistance, *R_f_* is the irreversible resistance, and *R_g_* is the gel layer resistance. Viscosity is explicitly presented in Darcy’s law. Here, it increases with solute concentration and decreases with temperature. At the same time, if the membrane is sensitive to temperature changes, this must be considered in Darcy’s law’s membrane resistance term [127]. According to our literature review, these models are the most used and reported. The main differences among them are the form in which the different resistances are analyzed and quantified for the prediction of permeate flux [1,46,83]. Table 3 shows the most relevant predictive models (based on number of citations and validations) published in the literature based on the resistance in series.

Chudacek and Fane [135] were the first authors in publishing a model related to resistance-in-series to predict permeate flux in a stirred-cell, considering a back-diffusion constant and a convective transport tested with colloid matrices such as silica sol and dextran albumin. This model was the origin of several investigations in stirred systems [122,147,152,153].

Conversely, it should be mentioned the role of the Hagen–Poiseuille equation to calculate the permeate flux, which is a physical law that describes the pressure drop in an incompressible and Newtonian fluid in laminar flow flowing through a long cylindrical pipe of constant cross-section. Namely, this equation illustrates how the permeate flux, through a microporous membrane, can be related to the number, diameter, and length of the pores, the pressure difference exerted across the membrane and the viscosity of the fluid [71]. The Hagen–Poiseuille model can be obtained from the integration of the Navier–Stokes equation [46,133], and it has been used for the modeling of the UF process with different matrices [71,132,133,134]. Gekas et al. [136] used this model to study the interaction of proteins and their absorption on the surface of polyacrylonitrile (PAN) UF membranes using BSA at constant pressure [121]. These authors found that the resistance attributed to the concentration polarization was responsible for the permeate flux drop reaching values two times higher than the hydraulic membrane resistance.

In 1997, De et al. [137] proposed a model that describes fouling as a boundary layer (based on film theory) composed of low molecular weight particles and a gel layer comprising the high molecular weight particles. The gel layer acts as a porous barrier, and it is considered a resistance in this model [154,155,156]. The model has been validated with different matrices such as orange juice [61], sucrose [137], stevia extract [157], and industrial wastewater [141]. Subsequently, Paris et al. [142] reported that models based on osmotic pressure and concentration polarization do not predict the permeate flux drop well since the permeate flux and concentration of molecules on the membrane surface vary along the membrane length, which is not considered in the mentioned models. Therefore, they propose a new model based on resistances, which included diffusive and convective transports in the equation [142]. Mohammadi et al. [146] carried out a new resistance model applied for oily wastewater emulsions as a modified version of the cake layer filtration model developed by Huang et al. [158] A good agreement between the model predictions and experimental data was found, especially at lower concentrations and lower transmembrane pressures. However, the model was not able to predict flux decline during the UF of gelatin suspensions.

In 2014, Sarkar et al. [159] published a semi-empirical model for a new cross-flow membrane module, named a radial flow membrane (RFM) module, which was tested in the UF of BSA with a flat disk polyethersulfone (PES) membrane having an MWCO of 30 kDa. The module was designed to ensure a smooth radial flow of the feed over the flat circular membrane. The module had a great fitting capacity in simulating the steady-state performance of the RFM module. However, it has a complex mathematical structure with just one parameter of adjustment.

The resistance-in-series models have been widely used, probably because the resistance concept is quickly understood when the membrane, acting as a barrier, rejects molecules with a higher molecular weight than the membrane pore size, creating an additional resistance on the membrane surface. Models included in Table 3 present the original and modified version of these models to improve the prediction capacity. Most of them showed a great ability to predict the permeate flux when tested with synthetic solutions. Nevertheless, the application to more complex matrices has not yet been investigated.

### 2.4. Fouling Models

These models have been mainly used for the identification of the type of fouling occurring in membrane filtration. However, these models can be used for the prediction of permeate flux. Based on cleaning techniques applied in membranes, fouling can be classified as reversible, i.e., the hydraulic permeability of the membrane can be recovered after a cleaning procedure; and irreversible, i.e., which is intended as the loss in the hydraulic membrane permeability [46,160]. Reversible fouling primarily occurs due to the loose bound external material placed on the membrane surface, which causes cake layer formation. In contrast, irreversible fouling may be caused due to strongly attached foulant components and pore-blocking of the membrane during membrane filtration. Membrane fouling has been recognized as one of the main drawbacks in membrane filtration; therefore, its minimization is crucial in any membrane application. Hermia [60] was the first one to develop a model describing the fouling mechanism in porous membranes for dead-end filtration. Hermia reformulated all models of blocking mechanism to a common power–law equation, as shown in Equation (7):

(7) $$\frac{d^{2}t}{dV^{2}}=k{\left(\frac{dt}{dV}\right)}^{n}\;$$

where *t* is the filtration time, *V* is the permeate volume, *k* is a phenomenological coefficient for dead-end filtration, and *n* is a general index which, depending on the fouling mechanism, assumes different values. In complete pore blocking (*n* = 2), the particle size is larger than the membrane pore size; thus, pores are completely blocked. In standard pore blocking (*n* = 1.5), particles are much smaller than the membrane pore diameter, so they can enter the pores and settle inside the pore walls, which may lead to pore blocking and pore volume reduction. In the intermediate blocking mechanism (*n* = 1), the particle size in the feed is the same as the membrane pore size; however, the membrane pore is not necessarily plugged by particles, and some particles may deposit on each other. Both large and small particles can accumulate on the membrane surface to form the cake layer in the cake formation mechanism (*n* = 0). This layer grows with time and causes future flux decline [5,14,46,161]. It should be noted that Hermia models have long been used to describe membrane filtration and fouling in constant transmembrane pressure. However, few studies have applied them to constant flux in a dead-end and cross-flow system, despite their frequent use of this mode of membrane operation in practical applications. Next, Field et al. [65] have discussed the relationship between constant-flux behavior and membrane fouling. They presented the concept of critical-flux, defined as the flux level where no fouling occurs. These authors mentioned that constant-flux filtration was obtained by moderately increasing transmembrane pressure. This operation method showed some advantages over normal constant-pressure filtration because it provides the possibility of avoiding over-fouling and reduces the severity of fouling. Even though Field et al. [65] were the first ones to present the critical flux concept, other authors previously mentioned a possible threshold flux when filtering a colloidal suspension [162]. These authors noted that permeate flux was higher compared with the expected flux from a balance between convection and classical dispersive forces (including diffusion, lateral migration, and shear-induced diffusion) in reverse osmosis experiments with ferric hydroxide. This behavior, attributed to surface interaction between colloidal particles, was called the colloid flux paradox. Field et al. [65] have indeed re-examined the Hermia model: they developed it for dead-end filtration, including a cross-flow mechanism. Later, Kirschner et al. [163] worked on a combined intermediate pore blocking and cake filtration model to describe fouling of a poly (ether sulfone) ultrafiltration with soybean oil emulsion.

New models that correlate the type of fouling with membrane performance in terms of permeate flux have been developed from Hermia’s model. Table 4 summarizes the most important models (based on the number of citations and validations) in the category of fouling and adsorption.

Ho and Zydney [66] developed a model capable of explaining filtrate flux data over the entire filtration process, accounting for both pore blockage and cake filtration. The model was verified using experimental data obtained during the constant pressure filtration of BSA through track-etched polycarbonate membranes over a range of bulk protein concentrations and transmembrane pressures. It was assumed that initial fouling was produced at the beginning of the filtration due to a pore-blocking phenomenon followed by a gel layer formation because of the growth of a protein cake or deposit over these initially blocked regions. Several authors have reported that the model developed by Ho and Zydney [66] showed a lower capacity of prediction in comparison with Hermia’s model. However, the model developed by Ho and Zydney [66] allows to determine the type of fouling using only one equation in comparison to Hermia’s model based on the use of four different equations (changing the n value of the constant) [66,176]. Alternatively, Mondal et al. [176] developed a model similar to that reported by Ho and Zydney [66], in which non-dimensional parameters are included for explaining the underlying principles of membrane fouling in the tested matrices, such as pineapple juice and deliming-bating of tannery effluents [176,177].

Fouling models have a similar structure to that of concentration polarization or resistance in series models. In addition, they have been mainly applied to determine the fouling mechanism occurring under defined operating conditions. Hermia’s and other models refer to only one type of fouling; however, in complex systems, more than one type of fouling can co-occur.

### 2.5. Non-Phenomenological Models

Non-phenomenological models comprise those entirely empirical, semi-empirical, and statistical in nature. Even though these kinds of models do not allow us to understand the different phenomena occurring in the membrane during filtration and relate them with operational parameters, in some cases, these models produced better results in terms of prediction capacity when compared to phenomenological models. In this context, Koltuniewicz et al. [179] indicated that the application of film theory is limited in cases where the wall region takes a predominant role in mass transport. In comparison, the semiempirical surface renewal model does not involve any particular interpretation of permeate flow resistance. Consequently, flux decline resulting from solute accumulation at the membrane surface, and therefore, the surface renewal rate, should be determined from the experiment on cross-flow UF. The authors mentioned that the use of the surface renewal model has advantages such as avoiding problems related to the diffusive or non-diffusive nature of the solute, osmotic, gel or some other factor or phenomenon that hinders the flux. Furthermore, the membrane or layer plays a predominant role in flow resistance. This model was experimentally validated using BSA solutions and kaolin suspension [179]. Finally, other applications of semi-empirical models have been used to understand the blocking mechanism in membrane filtration [59,68]. Regarding empirical models, the terms included in the equations do not have a physical meaning. Therefore, the relationship between operating parameters or membrane characteristics with membrane fouling cannot be deduced. However, the majority of these models guarantee a high capacity of permeate flux prediction [5,68,178]. Recently, Ohanessian et al. [81] proposed hybrid models to evaluate the performances of UF for the treatment of CMP effluents where the models were able to predict the filtration number of cycles adjustable according to the permeability recovery rate after physical washes of the membrane, the duration of physical and chemical washes and many operating parameters such as the transmembrane pressure, the nanoparticles concentration, the temperature, and the tangential velocity (for crossflow mode) [81]. Additionally, several efforts have been made in the modeling of permeate flux based on the analysis of phenomenological data [7,15,73,180] obtained experimentally, avoiding the use of specific transport mechanisms [69]. Among them, artificial neural networks (ANNs) have been applied in the field of membrane science and in other areas, including marketing, accounting, finance, health and medicine, engineering, and manufacturing [181,182,183]. One of the significant advantages of ANNs is their ability to learn and correlate input (operating conditions or membrane characteristics) to output (variables used to determine the membrane performance as permeate flux) data by pattern recognition of data without the necessity of understanding the process phenomena [184]. In this regard, specific applications have compared ANNs with a modified Hermia’s model to predict the permeate flux [72], the use of ANNs for predicting permeate flux and solute rejection in optimized polymers membranes [185], and the optimization and permeate flux control in wastewater treatment using a modification of Darcy law combined with ANNs [12].

Other strategies, such as the use of Response Surface Methodology (RSM), have been tested. In particular, the identification of the optimal operating conditions to simultaneously maximize the permeate flux and minimize the fouling index during the clarification of orange press liquor by UF has been applied [186]. Another example is the use of the Partial Least Square regression (PLSR) model to study the relationship among membrane characteristics, operating conditions, and membrane performance in terms of permeate flux and membrane rejection towards hesperidin and sugars (glucose, fructose, and sucrose) during the UF of orange press liquor [187]. PLSR has also been applied to investigate flux decline in a multi-stage ultrafiltration process [188]. Other authors have used Box-Jenkins Autoregressive Integrated Moving Average (ARIMA) modeling to forecast permeate flux in UF of fruit juices [69]. ARIMA models are essentially an exploratory data-oriented approach that has the flexibility of fitting an appropriate model, which is adapted from the structure of the data itself. With the aid of autocorrelation functions and partial autocorrelation functions, the time series’s stochastic nature can be approximately modeled, from which information such as trend, random variations, periodic component, cyclic patterns, and serial correlation can be discovered. As a result, forecasts of the series’s future values, with some degree of accuracy, can be readily obtained. This model is well established in the statistical literature with applications in several fields, such as economic forecasting, sales forecasting, budgetary analysis, stock market analysis, yield projections, process and quality control, inventory studies, workload projections, utility studies, and census analysis, with successful results. ARIMA forecasts the permeate flux with a prediction higher than 99.14% for the studied fruit juices [69]. Table 5 depicts a summary of non-phenomenological models (based on the number of citations and validations) reported in the literature for the prediction of permeate flux.

The quantity and description of all the model’s categories (phenomenological, empirical, and semi-empirical models) could lead the reader to two big questions: First, which model is convenient for a particular application? And second, what kind of models have a better performance for permeate flux prediction? A comparison between the models’ performance could contribute to identifying specific advantages and drawbacks for each model category to answer these questions.

## 3. Analysis of Model Goodness-of-Fit

The categories described previously comprised phenomenological, empirical, semi-empirical, and non-phenomenological models (e.g., statistical tools) developed between 1961 and 2019. In order to compare the capacity of permeate flux prediction, some models for each category were selected and tested with data of three fruit juices clarified by UF. The criteria used for the model selection include a series of items in the following order of importance:

(i) Type of configuration: models tested or developed for cross-flow filtration of fruit juices were selected.
(ii) Validation: models with more than one validation were considered.
(iii) The number of citations: models with a high number of citations were selected in order to take into account the scientific impact of each model.
(iv) Membrane module: models tested or developed in fruit juice processing with hollow fiber and tubular membranes were selected.
(v) Mathematical complexity: Considering the easy application of the models, the most straightforward models were preferred.

Based on these criteria, the models selected were: Shear-induced diffusion by Davis [57] for concentration polarization category; models described by Keden and Katchalsky [112] and Wijmans et al. [111] were selected for osmotic pressure; Hagen-Poiseuille and Boundary gel law described by De et al. [137] were selected for the resistance-in-series category; models described by Ho and Zydney [66], Mondal et al. [176] and the dynamic model by Song [108] were chosen for the fouling category; and models described by Yee et al. [191] and Ruby-Figueroa et al. [69] were selected within the non-phenomenological category. Simulations were performed using experimental data obtained in the UF of three different fruit juices processed for 10 h, as reported by Ruby-Figueroa et al. [69] In Table 6, characteristics of the juices, membrane types, and operating conditions are reported. Variables such as viscosity, bulk concentration, permeate volume, osmotic pressure, the resistance of the polarized layer, gel concentration, and gel thickness were obtained using a series of correlations available in the literature.

The determination of the quality of fit for the selected models was performed using the root mean square error (RMSE), the mean absolute percentage error (MAPE), and the percentage of variability explained (R2) at 95% confidence level. In addition, a validation procedure was carried out using residual analysis. The analysis of residuals, intended as the difference between the observed and predicted value, is fundamental for validating any model. The residuals represent the prediction error: they must have a random distribution and they must be unpredictable, which means that they must follow a normal distribution. In cases where the residuals do not have a normal distribution, the constants and predictors included in the model are intended not to be enough to predict the response. In this sense, two statistics, such as the Shapiro–Wilks (S-W) and Kolmogorov–Smirnov tests (KS), were used for determining the normal distribution of the residues for the analyzed models. Thus, it is expected that a valid model must demonstrate a normal distribution in at least one of the statistics used. All the computations were performed in Statgraphics Centurion XVI (Statgraphics Technologies, The Plains, VA, USA) and Excel 2010 (Microsoft, Redmond, WA, USA).

## 4. Results and Discussion of Selected Models’ Performance

Results obtained from the simulation of the models selected for each category are divided into three different sections according to the different juices studied (bergamot, kiwifruit, and pomegranate). In each section, a comparison between the models’ performance is specifically addressed.

### 4.1. Models’ Performance in Bergamot Juice Clarification

**Figure 2:**
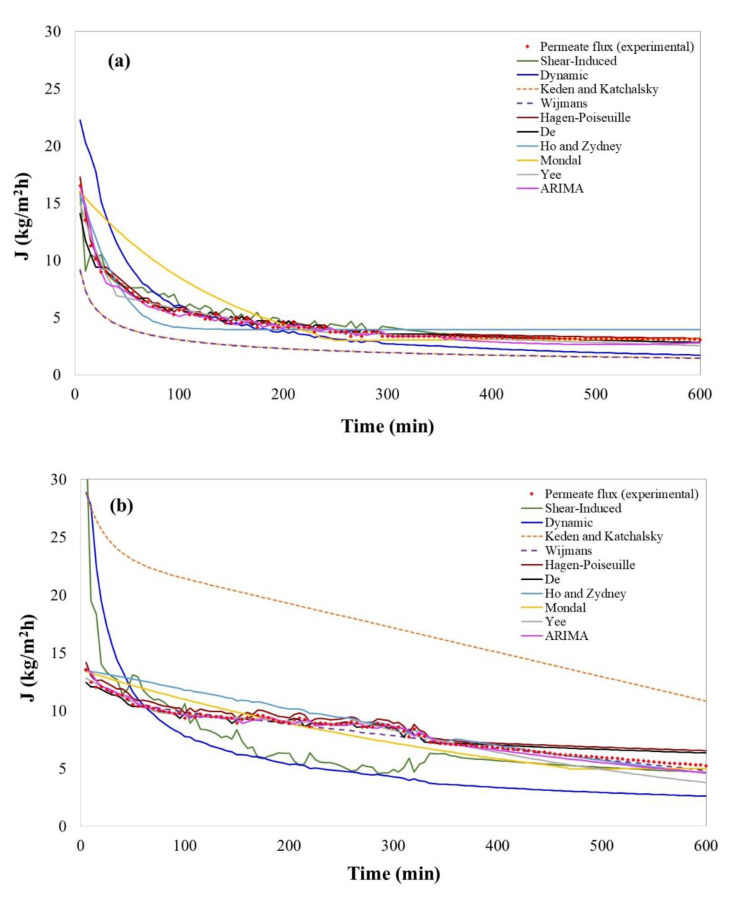

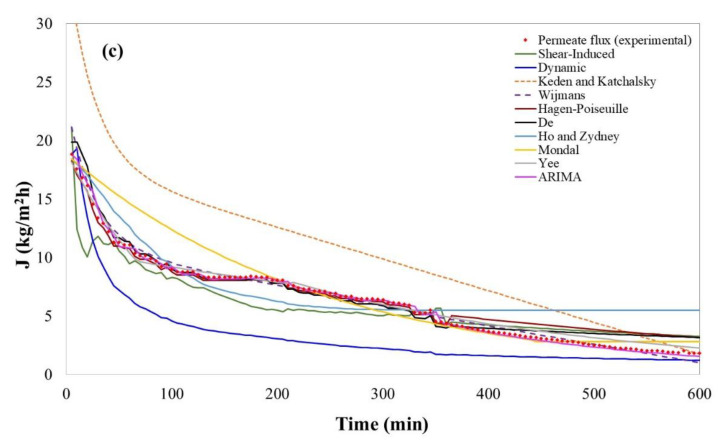
Permeate flux evolution obtained experimentally and predicted values by selected models for fruit juice clarification. (**a**) Bergamot juice; (**b**) kiwifruit juice; (**c**) pomegranate juice.

Figure 2a shows the time evolution of the permeate flux for bergamot juice clarification with respect to the predicted values of selected models. The flux-time curve is characterized by a rapid drop of the permeate flux from the initial value of 13.5 kg/m^2^h in the first 10 min of operation, followed by a long period of gradual flux decrease (until 190 min of operation) that ended with a steady-state flux of about 4 kg/m^2^h. Table 7 shows the results obtained for the statistical validation, in which more than one model was validated with *p*-values higher than 0.05 in at least one of the statistics used. According to Conidi et al. [27], the rapid decline of permeate flux in the UF of bergamot juice was attributed to gel-layer formation phenomena. This is confirmed by the results obtained in this work where the shear-induced model [57] showed 91.08% in the R-squared and lowered RMSE and MAPE (Table 7). Thus, it is evident that for bergamot juice, the gel-layer formation is confirmed. Based on the shear-induced model results, it can be deduced that the hydrodynamic diffusion of these particles occurs because they, individually, move randomly thanks to the current caused by the cut-off of the flow. Thus, the shear-induced model [57] assumes that after some time, the tangential flow compensates the convective transport of solutes on the surface of the membrane, preventing the growth of the gel-layer, and leading to a stationary value, as illustrated in Figure 2a. It should be mentioned that the shear-induced model assumes that the predominant mechanism is gel-layer formation, neglecting the existence of pore blockage. This may explain the goodness-of-fit obtained for this model: 91.08% (R-square), but not higher. Vincent Vela et al. [70] reported that the shear-induced model loses capacity of prediction at high cross-flow velocity (>2 m/s) and Δ*P* higher than 1 bar, a fact that was not corroborated in this work since the operating conditions were lower than the limits reported by Vincent Vela et al.

Even though concentration polarization models were developed to quantify the cake layer on the membrane surface and not for the direct quantification of permeate flux, the shear-induced model [57] is suitable for permeate flux prediction operated at low velocities and Δ*P*.

Besides, models in the osmotic pressure category, generally neglected in UF filtrations, such as the models developed by Keden and Katchalsky [112] and Wijmans et al. [111], were statistically validated. They showed a high capacity of prediction, which were higher than 99.17% in the R-square, and had lower values of RMSE and MAPE (Table 7). These models demonstrate that, in the case of bergamot juice, osmotic pressure should not be neglected. In this case, osmotic pressure was calculated, obtaining values in the range of 0.867–0.975, 0.674–0.724, and 0.300–0.584 bar for bergamot, kiwi, and pomegranate, respectively. The above confirms the impact on the permeate flux drop, which agrees with some authors who mentioned that in solutions with high concentrations, as in the case of bergamot juice, the osmotic pressure increases exponentially [124]. In addition, it has been reported that osmotic pressure limits permeate flux in membranes with a MWCO between 10 and 100 kDa [90]; however, osmotic pressure could be high for membranes retaining solutes smaller than 1 kDa [51]. Therefore, in membranes with MWCO of 100 kDa, as those used in the clarification of bergamot juice, osmotic pressure could have a considerable impact. Although osmotic pressure was not higher than Δ*P*, it cannot be neglected. Indeed, the osmotic pressure effect depends on the formation of the CP layer on the membrane, which increases the intrinsic concentration on the membrane surface. Thus, the assumptions related to whether the osmotic pressure is significant or not will depend on the type of matrix and operating conditions rather than on the membrane pore size. In particular, the empirical model tested by Keden and Katchalsky [112] showed an adequate capacity of prediction for the permeate flux (99.17% in R2 and lower RMSE and MAPE). This model is one of the most widely cited models and it has been the basis for many other models [113,114,115,116,117]. In this context, Matos et al. (2016) used it successfully to purify oil–water emulsions with a ceramic tubular membrane, having an active layer outside the membrane [116]. Similar to the model of Keden and Katchalsky [112], the boundary layer resistance (BLR) model developed by Wijmans et al. [111] showed better goodness-of-fit for the bergamot juice (99.22% in R2 and lower RMSE and MAPE). This model particularly integrates osmotic pressure with the concept of a boundary layer resistance [80]. In this regard, and in cases where the process operates at high ΔP, the existence of a gel layer and the formation of a new secondary dynamic membrane on the first structure can certainly occur [2]. Thus, when there is an increase in the concentration (e.g., in batch concentration mode processes), the boundary layer becomes a hydrodynamic resistance for solute permeation [119]. The Wijmans et al. (1984) model [111] was validated by Cheng et al. [80], using a hollow fiber membrane with Dextran T500 in aqueous solution. This model showed better goodness-of-fit in comparison to Keden and Katchalsky’s model in permeate flux prediction of bergamot juice. This can be attributed to the fact that Wijmans et al. (1984) model is semi-empirical compared to the model by Keden and Katchalsky, which is entirely empirical.

Although both models were developed and validated with model solutions as BSA and PEG, osmotic pressure correlation is also available in the literature. However, a good performance was observed for a complex matrix such as in the bergamot juice.

Non-phenomenological models, such as those by Yee et al. [191] and Ruby-Figueroa et al. [69], were statistically validated and showed an adequate capacity of prediction. In particular, the ARIMA model showed an R^2^ of 97.92 and lower values of RMSE and MAPE.

In contrast, models developed by Ho and Zydney [66], Mondal et al. [176] and the dynamic model [108] also included in the fouling model category, were not statistically validated. This means that the constant and variables involved in both models are not enough to predict the permeate flux in the bergamot juice filtration by UF.

### 4.2. Models’ Performance in Kiwifruit Juice Clarification

Figure 2b shows the permeate flux evolution, as well as the simulation results of the selected models for the prediction of permeate flux, in the clarification of kiwi juice. Permeate flux decreased 61% after 600 min of operation. The decline in flux after the first 100 min of operation is less pronounced until ~300 min; after that, a drastic decline is observed. This variability of the permeate flux with time is a tough challenge for modeling. Table 8 shows the simulation results for the selected models in which the categories such as osmotic pressure, resistance-in-series, fouling and adsorption, and non-film theory models showed an adequate capacity of permeate flux prediction.

In this case, the concentration polarization model was not validated and showed lower values for prediction capacity (R^2^ ≤ 52.86). Concentration polarization models assume that the cake layer, once it is formed, remains constant. In this case, this is not appreciated since the drop in permeate flux was observed along the whole process.

In particular, the resistance-in-series models developed by De et al. [137] showed a good capacity of prediction with high R^2^ (97.43%) and lower values of RMSE and MAPE, as shown in Table 8. This model is based on a convective transport with respect to the driving force in a porous medium in which separation occurs by size exclusion [4,46,197,198]. Despite the high capacity of fit for this model, R-square could be higher if some changes are made in the equation, such as some modifications in the Leveque relationship (used for the determination of mass-transfer coefficient) [100,199,200,201], which includes the gel-layer growing [202,203] or the viscous effect, and modification in the Sherwood correlation to improve the capacity to determine the mass-transfer and the thickness of the gel layer with a higher precision [61,204].

The Hagen–Poiseuille model has been applied to predict permeate flux in the treatment of oil industry wastewater at a pilot scale [71] and solvents such as water and ethanol in ceramic membranes by MF and UF [133], showing a good prediction capacity. However, for the kiwifruit juice, the model was not validated statistically. This result can be attributed to some factors not included in the Hagen-Poiseuille model, such as capillarity or dipole, which could affect kiwifruit juice’s prediction capacity.

Models in the category of fouling and adsorption, such as the one developed by Ho and Zydney [66], showed a good prediction capacity for kiwifruit juice with high R^2^ and lower values of RMSE and MAPE (Table 8). This model assumes that an initial blocking step exists on the membrane surface, followed by the development of a gel layer in a steady-state [66,176]. The model has also been validated with a BSA solution [66], protein solution [205], wastewater containing oil [206], and concentrated protein solution [207] with good goodness-of-fit.

Regarding the category of non-phenomenological models, the two tested models showed great goodness-of-fit (Table 8). In particular, the model developed by Yee et al. [191], which was validated with milk whey, showed a great goodness-of-fit during the first 2.5 h of operation. Similar results were obtained in the present work, where the capacity of fit was lower after 300 min of operation. Thus, the use of this model for a long-term forecast is questionable. Alternatively, the ARIMA model [69] showed the highest values of R^2^ (98.98%) and lower values of RMSE and MAPE. Thus, non-phenomenological models appear to be the most adequate for permeate flux curves with high variability.

### 4.3. Models’ Performance in Pomegranate Juice Clarification

The time evolution of permeate flux for pomegranate juice is shown in Figure 2c; in this case, a continuous drop of permeate flux from 13.38 kg m^−2^h^−1^ to 1.81 kg m^−2^h^−1^ after 600 min of operation can be observed. The absence of a stationary point implies difficulty for modeling. In addition, the filtration of pomegranate juice was carried out at low ΔP and a high cross-flow velocity. According to several authors, these conditions lead to a loss in the capacity of prediction of some models [58,61,168,203]. The validation of the selected models for pomegranate juice in terms of permeate flux prediction is shown in Table 9.

The model developed by Ho and Zydney [66], included in the category of fouling and adsorption, had the lowest R-square (75.91%) within the validated statistical models. Some phenomenological models were validated from a statistical point of view; however, they showed a lower capacity of prediction in comparison to non-phenomenological models (Table 9). In particular, the model developed by Davis [57] showed limitations in the prediction after 240 min of the process with an R-square of 85.58% and a high value of MAPE.

The non-phenomenological models showed the highest prediction capacity for the permeate flux in the UF of pomegranate juice, with R-squares higher than 99.20% and lower values of RMSE and MAPE. Thus, similar to kiwifruit and bergamot juice, these kinds of models appear to be adequate for the prediction of permeate flux. However, it is worth noticing that these kinds of models do not allow the understanding of the mechanism of fouling or the identification of the effect of some operating conditions on the permeate flux. In particular, the ARIMA model is characterized by high adaptability to different data structures through autocorrelation and partial autocorrelation [208], which were ratified with the highest R-squares (99.70%) within the validated models for pomegranate juice filtration. On the contrary, the model developed by Yee et al. [191] showed a good capacity for prediction (R^2^ 99.2%). It is particularly interesting that the ARIMA model and the one reported by Yee et al. [191] have been validated for more than 10 h of operation with a great capacity of forecast [171,191,209,210]. Thus, both models appear to be adequate for the long-term prediction of permeate flux.

## 5. Conclusions

Permeate flux prediction is an essential parameter in membrane performance evaluation and the projections for scaling-up from laboratory to the pilot plant or the industrial scale. This work includes a critical review and analysis of the most cited and validated models for predicting permeate flux in UF for the 1961–2019 period. These models were grouped into two categories: phenomenological (comprising four types of models such as gel-polarization, osmotic pressure, resistance-in-series, and fouling models) and non-phenomenological models. Ten models (two for each type of model) were selected for a careful comparison, including statistical tools, of the prediction capacity. The capacity of prediction was validated by comparing the predicted values of each model with experimental data of three fruit juices: bergamot, kiwi, and pomegranate. Results of statistically validated models showed high variability in the prediction capacity by phenomenological models for the studied juices. In particular, phenomenological models present a capacity of prediction ranging from 75.91 to 99.78% (R-squares), whereas the Mean Absolute Percentage Error (MAPE) ranged from 3.14 to 51.69, and Root Mean Square Error (RMSE) from 0.22 to 2.01. Non-phenomenological models showed a better prediction of permeate flux with R-squares higher than 97% and lowered MAPE (0.25–2.03) and RMSE (3.74–28.91) in comparison with phenomenological models. The majority of the phenomenological models were developed and validated with model solutions such as BSA, PEG, and dextran. However, some of them lost prediction capacity in complex matrices where the model’s assumption appeared not to be enough. Despite this situation, some phenomenological models such as those developed by Wijmans et al. (1984) and De et al. (1997), as well as non-phenomenological models such as Yee et al.’s (2009) and Ruby-Figueroa et al.’s (2017), showed a good capacity of prediction and lower values of RMSE and MAPE for the three investigated fruit juices. Considering that non-phenomenological models showed better results in terms of prediction, the reader may tend to choose these models; however, they do not give any information related to the effect of different parameters on the permeate flux, a crucial point for the system scaling-up. In this regard, the non-phenomenological models are an excellent prediction tool of permeate in well-established operations with limited variability in the feed matrix characteristics. On the contrary, phenomenological models are still a proper method for scaling-up purposes, mainly for research in the understanding of the UF process. Therefore, the challenge herein is the development of new phenomenological models with assumptions that include the different phenomena occurring in the filtration of complex matrices in order to improve the capacity of prediction of permeate flux in long-term operation.

## Figures and Tables

**Table 1:** Summary of the relevant models for the concentration polarization category, including the Boundary layer, polarized concentration, and gel models.

No.	Model	Authors	Ref.	Validation Matrix	Main Transport Mechanism	Configuration	Module Type	Number of Citations	Model Validation in Publications
(1.1)	$J=\frac{D}{\delta }\ln\frac{C_{g}}{C_{b}}=k\ln\frac{C_{g}}{C_{b}}$	Film theory	[46]	-	Diffusive	Cross-flow	-	-	[14,89]
(1.2)	$J={\left(D/\pi t\right)}^{1/2}\;\ln\left[\frac{c_{g}-c_{p}}{c_{o}-c_{p}}\right]$	Trettin and Doshi (1980)	[62]	BSA	Diffusive	Dead-end	Unstirred cell	76	-
(1.3)	${J=x}_{1}k\ln\left(\frac{C_{g}}{C_{b}}\right)+\left({1-x}_{1}\right)\;k\ln\left(\frac{C_{g}}{C_{b}}\right)$	Modified gel-polarization Fane et al. (1981)	[87]	Gamma Globulin BSA	Diffusive-Convective	-	-	164	-
(1.4)	$J=0.078{\left({\frac{rp}{L}}^{4}\right)}^{1/3}\gamma \ln\left(\frac{C_{g}}{C_{b}}\right)$	Zydney and Colton (1986)	[63]	Blood	Diffusive	Cross-flow	-	274	-
(1.5)	J=ΔPμRm1+2β(t-D(Cgv-Cov-Covln(CgCo))(ΔPμRm)2)Cg-CoCodh2ΔPμRm	Shear-induced diffusion Davis (1992)	[57]	PEG	Diffusive-Convective	Cross-flow	Tubular	158	[70]
(1.6)	$J=\frac{\Delta P}{L\beta v{\left(L\right)}^{2}}\left[1-\frac{L_{p}}{\Delta P}v\left(L\right)\right]$	Song and Elimelech (1995)	[90]	-	Diffusive-Convective	Cross-flow	Rectangular channel	246	[91]
(1.7)	$J\cdot 6\pi \mu_{0}\cdot \sum_{i=0}^{\infty }f_{i}\emptyset^{i}=-kT\frac{{\left(1-\emptyset \right)}^{3}}{\left(\emptyset -\emptyset_{p}\right)}(1+\overset{\infty }{\underset{i=2}{\sum }}A_{i}\emptyset^{i-1})\frac{d\emptyset }{dx}$	Jonsson and Jonsson (1996)	[92]	Silica sol	Diffusive-Convective	Cross-flow	-	71	-
(1.8)	$J=\frac{2\pi \int_{0}^{b}V_{f}\left(r\right)\;r\;\mathrm{dr}}{{\pi b}^{2}}$	Saksena and Zydney (1997)	[93]	BSA and IgG	Diffusive-Convective	Dead-end	Stirred cell	51	-
(1.9)	$JH/D_{o}=-{\left[\frac{{\left(1-bc_{o}\emptyset \right)}^{6.5}}{\emptyset -\emptyset_{p}}\frac{\partial \emptyset }{\emptyset \eta }\right]}_{\eta =0}$	Bhattacharjee and Datta (1991)	[94]	PEG-6000	Diffusive-Convective	Dead-end	Unstirred cell	9	-
(1.10)	$J_{t}\left(t\right)=\left(J_{0}-J_{\infty }\right)e^{-\frac{t}{t_{0}}}+J_{\infty }$	The relaxation model Konieczny (2002)	[95]	Water potable	Diffusive-Convective	Cross-flow	Tubular	22	[96]
(1.11)	$\bar{J}=0.807{\left(\frac{\gamma_{w}}{L}{\left(\frac{KT}{3\pi \mu D_{m}}\right)}^{2}\right)}^{1/3}ln\frac{c_{g}}{c_{o}}$ Model parameter: $\gamma_{w}$	Neggaz et al. (2007)	[97]	Pectin Albumin	Diffusive-Convective	Cross-flow	Hollow fiber	6	-
(1.12)	$J=\frac{\Delta {P-2502\;C}_{o}}{{1.79\;\times \;10}^{13}\mu +{9.327x10}^{14}C_{o}r_{p}^{2/3}}$; Brownian diffusion J=ΔP-2502 Co1.79 × 1013μ+62.73Corp43; Shear induced diffusion $J=\frac{\Delta {P-2502\;C}_{o}}{{1.79\;\times \;10}^{13}\mu +\frac{{340C}_{o}}{{\left[12.639r_{p}^{2}+\frac{{2.204\times 10}^{-19}}{a}\right]}^{2}}}$; Combined diffusion	Singh et al. (2013)	[64]	Synthetic Fruit juice	Diffusive-Convective	Cross-flow	Spiral-wound	10	-

BSA: Bovine serum albumin; PEG: Polyethylene glycol; IgG: Immunoglobulin G.

**Table 2:** Summary of developed models for permeate flux prediction in which osmotic pressure is considered.

No.	Model	Authors	Ref.	Validation Matrix	Main Transport Mechanism	Configuration	Module Type	Number of Citations	Model Validation in Publications
(2.1)	$J=\frac{\left|\Delta P\right|-\left|\Delta \pi \right|}{{\mu R}_{m}}$	Osmotic pressure Keden and Katchalsky (1958)	[112]	Water	Convective	Dead-end	-	442	[113,114,115,116,117]
(2.2)	J=A(ΔP-Δπ)	Goldsmith (1971)	[118]	Dextran fractions (polysaccharides)	-	Cross-flow Dead-end	Tubular Stirred cell	138	-
(2.3)	$J=\frac{\Delta P-ac_{b}^{n}\exp(nJ/k)}{R_{m}}$ Model parameters: a, n	Wijmans et al. (1984)	[111]	-	-	-	-	201	[80,119]
(2.4)	$R_{P}\left(t\right)=\frac{R_{ma}}{1-\frac{\sigma \Delta \pi_{m}}{\Delta P}}-R_{ma}=R_{ma}\left(\frac{J_{w}}{\upsilon_{w}}-1\right)$	Bhattacharjee and Bhattacharya (1992)	[58]	BSA	Convective	Dead-end	Unstirred cell	36	[17]
(2.5)	$J=\frac{\Delta P-\Delta \pi }{\left[R_{m}+\left(V/A-J^{*}t\right)\alpha \left(c_{b}-c_{p}\right)\right]\mu }$ Model parameter: α	Bhattacharjee and Bhattacharya (1992)	[25]	PEG	Convective	Dead-end	Unstirred cell	50	-
(2.6)	$J=\frac{\upsilon_{p}^{o}}{{1+R}_{\mathrm{ps}}^{*}\left[{1-e}^{\left({-K}_{1}t\right)}\right]}$ Model parameters: $R_{\mathrm{ps}}^{*}$, K_1_	Bhattacharya et al. (2001)	[21]	Sugar cane	Convective	Dead-end	Stirred cell	42	[120]
(2.7)	$J=\frac{\Delta P-\Delta \pi_{i-1}}{R_{m\;i-1}+\left(\frac{R_{m}^{*}\Delta t}{t_{R}}\right)+\left(\frac{m_{i}-\Delta t}{J_{i-1}}\right)}$ Model parameters: *t_R_*, *m_i_*	Kanani and Ghosh (2007)	[121]	HSA	Convective	Dead-end	Stirred cell	28	-
(2.8)	$J=\frac{\Delta P+\frac{\sigma RT}{V_{1}}\left[\ln\left(\frac{\rho_{pol}-C_{m}}{\rho_{pol}-C_{p}}\right)+\left\{\left(1-\frac{1}{n}\right)+X_{12}\frac{C_{m}+C_{p}}{\rho_{pol}}\right\}\frac{C_{m}-C_{p}}{\rho_{pol}}\right]}{\mu R_{m}}$ Model parameters: α, *n*, *X*_12_	Sarkar et al. (2010)	[122]	PEG-6000	Diffusive-Convective	Dead-end	Stirred cell	2	-
(2.9)	${J=k}_{o}{\left(\frac{\mu_{b}}{\mu_{o}}\right)}^{1/3}\int_{C_{b}}^{C_{w}}\left(\frac{\mu_{o}}{\mu }\right)\left(\frac{M_{p}}{RT}\right)\left(\frac{d\Pi }{dC}\right)\frac{dC}{C}$	Binabaji et al. (2015)	[123]	Protein solution	Diffusive	Cross-flow	Tangential flow filtration (TFF) Cassette	6	-

HSA: Human serum albumin solution; BSA: Bovine serum albumin; PEG: Polyethylene glycol.

**Table 3:** Summary of resistance-in-series models developed for permeate flux prediction.

No.	Model	Authors	Ref.	Validation Matrix	Main Transport Mechanism	Configuration	Module Type	Number of Citations	Model Validation in Publications
(3.1)	$J=\frac{\left|\Delta P\right|}{\mu R_{t}}$	Resistance Darcy’s law	-	-	Convective	Dead-end Cross-flow	Tubular	-	[12,23,128,129,130,131]
(3.2)	$J=\frac{\varepsilon d_{p}^{2}\Delta P}{32\Delta x\mu }$	Hagen-Poiseuille	-	Solvent	Convective	Dead-end Cross-flow	Tubular	-	[71,132,133,134]
(3.3)	$\frac{1}{A}\frac{dV}{dt}=\frac{\Delta P}{\left[R_{m}+\left(V/A-J_{ss}t\right)\alpha C_{b}\right]\eta }$ $J_{ss}=k\;ln\left(C_{g}/C_{b}\right)$ Model parameters: *J_ss_*, α	Agitation resistance Chudacek and Fane (1984)	[135]	Silica sol Albumin Dextran	Convective	Dead-end Cross-flow	Unstirred cell	167	-
(3.5)	$J=\frac{\Delta P-\Delta \Pi }{\mu \left(R_{m}+R_{g}\right)}$ $R_{m}=\Delta P/J_{o}$ $R_{ac}^{*}=\left(J_{o}/J_{f}\right)-1$ $R_{m}+R_{ad}=\Delta P/J_{f}$	Adsorption resistance Gekas et al. (1993)	[136]	BSA	Convective	Cross-flow	Plate type	44	-
(3.6)	$J=\frac{\Delta P-\Delta \Pi }{\mu \left(R_{m}+R_{g}\right)}$ $R_{g}=\alpha \left(1-\epsilon_{g}\right)\rho_{g}L$ $\alpha =180\frac{\left(1-\epsilon_{g}\right)}{\epsilon^{3}d_{p}^{2}\rho_{g}}$	De and Bhattacharya (1997)	[137]	Mixture of sucrose and poly(vinyl alcohol)	Diffusive-Convective	Cross-flow	Stirred cell	66	[61,131,138,139,140,141]
(3.7)	$J=\frac{1}{\mu \left(R_{M}+R_{cp}\left(z\right)\right)}\left(P\left(z\right)-P_{p}\right)$ $P(z)=\frac{P_{i}-P\left(z\right)}{z}=\frac{16}{Re}\frac{\rho u_{0}^{2}}{R}$	Paris et al. (2002)	[142]	Dextran T500	Diffusive-Convective	Cross-flow	Tubular	45	[143]
(3.8)	$\frac{1}{J}=\frac{\mu R_{m}}{\Delta P}+\frac{\mu }{P_{m}\Delta P}\left[\frac{V}{A}\left(\frac{c_{b}-c_{p}}{c_{g}-c_{b}}\right)-\frac{k_{b}}{A}\frac{c_{g}}{c_{g}-c_{b}}\omega t\right]$ Model parameters: *P_m_*, *k_b_*, *ω*	Bhattacharjee and Datta (2003)	[144]	PEG-6000	Diffusive-Convective	Dead-end	Stirred cell	31	-
(3.9)	$J_{mf}\left(z,t\right)=\frac{\Delta P\left(z,t\right)}{\mu \left(R_{mf}+R_{c}\left(z,t\right)\right)}=\frac{-p_{i}\left(z,t\right)}{\mu \left(R_{mf}+R_{c}\left(z,t\right)\right)}$	Chang et al. (2005)	[145]	Polystyrene latex	Convective	Dead-end	Hollow fiber	54	-
(3.10)	$J=\frac{\Delta PA\beta }{\mu }\frac{1}{{\left(V-V_{c}\right)}^{\frac{1}{\alpha }-1}}+\gamma$ Model parameters: *A*, β, γ	Mohammadi et al. (2005)	[146]	Emulsion of oil and gelatin	Diffusive-Convective	Cross-flow	Plate and frame	26	-
(3.11)	$J(z)=\frac{\Delta P\left(z\right)}{R_{m}+R_{f}+\phi \Delta P\left(z\right)}$	Yeh and Chen (2005)	[147]	Dextran T500	Convective	Cross-flow	Tubular	6	-
(3.12)	$1-\left(\frac{\bar{J}}{J_{lim}}\right)=e^{-{\left(\bar{\Delta P}\right)}_{exp}/\left(RJ_{lim}\right)}$	Yeh (2008)	[148]	Dextran T500	Convective	Cross-flow	Hollow fiber	8	-
(3.13)	$J=\frac{P_{TM}}{\eta_{perm}\left(R_{m}+R_{c}\right)}$ $R_{c\;}=\alpha \frac{m_{X}^{c}}{A}$ $\alpha =\alpha_{0}{\left(\frac{P_{TM}}{P_{TM,0}}\right)}^{n}$ Model parameters: *P_TM_*, *α*, $\alpha_{0}$	Cuellar et al. (2009)	[149]	*E. coli* cells	Convective	Cross-flow	Hollow fiber	7	-
(3.14)	$\bar{J}=\int_{0}^{1}\frac{-\Delta P_{i}d\varepsilon }{A\varepsilon_{2}+B\varepsilon +C}+\int_{0}^{1}\frac{(mQ_{i}-n\bar{J})\varepsilon \;d\varepsilon }{A\varepsilon_{2}+B\varepsilon +C}$ Model parameters: ℰ, *A*, *B*, *C*, *n*	Yeh et al. (2010)	[150]	Dextran T500	Convective	Cross-flow	Tubular	1	-
(3.15)	$J_{D}=\frac{1}{\left(R_{s}+\frac{R_{m}R_{p}}{R_{m}+R_{p}}\right)}$ $R_{s}=k_{s}\frac{r_{s}}{r_{p}}exp\left(1-\beta \right)$ $R_{m}=k_{m}\left(\frac{\mu }{\alpha }\right)$ $R_{p}=k_{p}{\left(\frac{r_{s}}{r_{p}}\right)}^{2}\mu$	Marchetti et al. (2012)	[133]	Water Ethanol Acetone DMF	Convective	Cross-flow	Tubular	37	-
(3.16)	$J=\frac{\Delta P}{\mu \left[R_{m}+\left(R_{ad,ss}+R_{cp,\;ss}\right)\left(1-e^{-bt}\right)+\left(\frac{m_{p}}{A}\right)\alpha \right]}$ Model parameters: σ, *b*	Corbatón-Báguena et al. (2018)	[151]	Whey model solution	Diffusive-Convective	Cross-flow	Tubular Flat sheet	6	-

DNA: Deoxyribonucleic acid; DMF: N, N-dimethylformamide; BSA: Bovine serum albumin; PEG: Polyethylene glycol.

**Table 4:** Summary of models developed for permeate flux prediction based on fouling and adsorption mechanisms.

No.	Model	Authors	Ref.	Validation Matrix	Main Transport Mechanism	Configuration	Module Type	Number of Citations	Model Validation in Publications
(4.1)	$1/J^{2}=1/J_{o}^{2}+k_{c}k_{cf}t;\;n=0$ $1/J=1/J_{o}+k_{i}t;\;n=1$ $\frac{1}{\sqrt{J}}=\frac{1}{\sqrt{J_{o}}}+K_{s}t;\;n=1.5$ $ln\;J=lnJ_{o}-k_{c}t;\;n=2$	Hermia (1982)	[60]	-	Convective	Dead-end	-	-	[13,14,164,165]
(4.2)	$\ln\left[\frac{{1-R}_{\mathrm{obs}}}{R_{\mathrm{obs}}}\right]=\ln\left[\frac{{1-R}_{m}}{R_{m}}\right]+\frac{J}{k}$	Nakao and Kinura (1981)	[114]	PEG	Convective	Dead-end	Tubular	32	[166]
(4.3)	$J_{p}{=J}_{\mathrm{pss}}+\left(J_{o}{-J}_{\mathrm{pss}}\right)e^{{-k}_{c}J_{o}t}$; Complete blocking $J_{p}=\frac{J_{o}J_{\mathrm{pss}}\left(e^{k_{i}J_{\mathrm{pss}}t}\right)}{J_{\mathrm{pss}}{+J}_{o}\left(e^{k_{i}J_{\mathrm{pss}}t}-1\right)}$; Intermediate blocking Jp=Jo(Jo+Jo12kst)2; Standard blocking $t=\frac{1}{k_{\mathrm{gl}}J_{\mathrm{pss}}^{2}}\ln\left[\left(\frac{J_{p}}{J_{o}}\frac{J_{o}{-J}_{\mathrm{pss}}}{J_{p}{-J}_{\mathrm{pss}}}\right){-J}_{\mathrm{pss}\left(\frac{1}{J_{p}}-\frac{1}{J_{o}}\right)}\right]$; Gel layer formation	Cros-flow HermianField et al. (1995)	[65]	Dodecane-water emulsion	Convective	Cross-flow	Flat-sheet	945	[9,167,168,169]
(4.4)	$Sh=\frac{1}{\left(W_{d}-1\right)e^{-P_{e}}+\frac{1}{P_{e}}\left(1-e^{-P_{e}}\right)}$	Bacchin et al. (1996)	[24]	Clay suspensions	Diffusive	Cross-flow	Hollow fiber	88	[6]
(4.5)	$J=\frac{1}{L}\left[\int_{0}^{x\left(t\right)}v_{eq}\left(x\right)dx+\left(L-X\left(t\right)\right)v\left(t\right)\right]$ When *t* < *t_ss_* $J=1.31{\left(D^{2}\gamma /L\right)}^{1/3}{\left(c_{g}/c_{0}-1\right)}^{1/3}$ When *t* > *t_ss_*	Dynamic model Song (1998)	[108]	-	Diffusive-Convective	Cross-flow	-	253	[45,55]
		Wang and Song (1999)	[170]	Silica colloids	Diffusive-Convective	Cross-flow	Tubular	62	-
(4.6)	$J=J_{o}\left[e^{\left(-\frac{\alpha \Delta Pc_{b}}{\mu R_{m}}t\right)+\frac{R_{m}}{R_{m}+R_{p}}\left(1-e^{\left(-\frac{\alpha \Delta Pc_{b}}{\mu R_{m}}t\right)}\right)}\right]$	Ho and Zydney (2000)	[66]	BSA	Convective	Cross-flow	Stirred cell	434	[10,171,172,173]
(4.7)	$J=Df\left(\partial \right)S_{D}\left(\partial \right)\frac{Ak}{\Delta x}\Delta C_{s}+\bar{JC_{s}}g\left(\partial \right)S_{F}\left(\partial \right)$ Model parameters: ∂, *S_D_*, *S_F_*	Darnon et al. (2002)	[172]	Β-Lactoglobulin and yeast extract	Diffusive-Convective	Cross-flow	Tubular	12	-
(4.8)	$V=\frac{J_{O}}{k_{b}}\left({1-e}^{\left(\frac{{-k}_{b}}{k_{c}J_{o}^{2}}\left(\sqrt{{1+2k}_{c}J_{o}^{2}t}-1\right)\right)}\right)$ Cake-complete $V=\frac{1}{k_{i}}\ln\left(1+\frac{k_{i}}{k_{c}J_{o}}\left({\left({1+2k}_{c}J_{o}^{2}t\right)}^{1/2}-1\right)\right)$ Cake-intermediate $V=\frac{J_{o}}{k_{b}}\left({1-e}^{\left(\frac{{-2k}_{b}t}{{2+k}_{s}J_{o}t}\right)}\right)$ Complete-standard $V=\frac{1}{k_{i}}\ln\left(1+\frac{{2k}_{i}J_{o}t}{{2+k}_{s}J_{o}t}\right)$ Intermediate-standard $V=\frac{2}{k_{s}}\left(\beta \cos\left(\frac{2\pi }{3}-\frac{1}{3}arccos\left(\alpha \right)\right)+\frac{1}{3}\right)$ Cake-standard Model parameters: *K_b_*, *k_c_*, *k_i_*, *k_s_*, *α*, *β*	Bolton et al. (2004)	[173]	IgG BSA	Convective	Cross-flow	Tubular	201	-
(4.9)	$\frac{Q}{Q_{o}}=\frac{1}{{\left({1+\beta Q}_{o}C_{b}t\right)}^{2}}e^{\left(-\frac{{\alpha C}_{b}J_{o}t}{{1+\beta Q}_{o}C_{b}t}\right)}+\;\int_{0}^{t}\frac{\left({\alpha C}_{b}J_{o}/{\left({1+\beta Q}_{o}C_{b}t_{p}\right)}^{2}\right)e^{\left(-\left({\alpha C}_{b}J_{o}t_{p}/\left({1+\beta Q}_{o}C_{b}t_{p}\right)\right)\right)}}{\sqrt{{\left[\left(R_{po}{/R}_{m}\right)+{\left({1+\beta Q}_{o}C_{b}t_{p}\right)}^{2}\right]}^{2}+2(f^{\prime }R^{\prime }\Delta pC_{b}/\mu R_{m}^{2}(t-t_{p})}}{dt}_{p}$ Model parameters: α, β, tp, f′, R′	Duclos-Orsello et al. (2006)	[174]	BSA	Convective	Dead-end	Stirred cell	152	-
(4.10)	$\frac{\mathrm{dJ}}{\mathrm{dt}}=-a\frac{C_{b}}{C_{\mathrm{bo}}}\left({J-J}^{*}\right)J^{2}$ $J^{*}{=J}_{\left(C_{\mathrm{bo}}\right)}^{*}{\left(\frac{C_{b}}{C_{\mathrm{bo}}}\right)}^{-n}$	Furukawa et al. (2008)	[67]	Soy less	Diffusive-Convective	Dead-end Cross-flow	Tubular	27	-
(4.11)	$J\left(t\right)=J\left(t\to \infty \right){+\mathrm{ke}}^{\left(-\mathrm{bt}\right)}$ Model parameter: b	Lin et al. (2008)	[175]	BSA Hemoglobin	Diffusive-Convective	Dead-end	Stirred glass cell	21	-
(4.12)	$J=\frac{J_{0}}{{1+R}_{\mathrm{CPB}}^{*}}$ $J_{t_{1}}=\frac{J}{{1+R}_{\mathrm{CPB}}^{*}t_{1}}$ Model parameter: $R_{\mathrm{CPB}}^{*}$	Mondal and De (2009)	[176]	Pineapple juice	Convective	Cross-flow	Hollow fiber	29	[177]
(4.13)	$\Delta P=\frac{\bar{J}}{k}$$\frac{k}{k_{o}}=\frac{{\left(1-\frac{\sigma }{\varepsilon_{o}}\right)}^{3}}{{\left[1+\sigma /\left({1-\varepsilon }_{o}\right)\right]}^{2}}$ Model parameter: σ	Wang et al. (2017)	[178]	Aqueous solutions	Diffusive-Convective	Cross-flow	Hollow fiber	1	-

BSA: Bovine serum albumin; PEG: Polyethylene glycol; IgG: Immunoglobulin G.

**Table 5:** Summary of non-phenomenological models used for the prediction of permeate flux.

No.	Model		Authors	Ref.	Validation Matrix	Main Transport Mechanism	Configuration	Module Type	Number of Citations	Model Validation in Publications
(5.1)	$\bar{J}=\frac{\left(J_{o}-J^{o})(J_{\lim}-J^{o}\right)}{J^{o}+J_{\lim}-J^{o}}$ Model parameter: *J*^0^		Surface renovation theory Koltuniewicz (1992)	[179]	BSA	Diffusive-Convective	Cross-flow	-	44	-
(5.2)	$J\left(t\right){=J}_{p,o}-\mathrm{at}\;\;J(t)\leq J_{\mathrm{th}}$ $J\left(t\right)=\left(J_{p,o}{-J}_{\mathrm{th}}\right)e^{-b\prime t}{+J}_{\mathrm{th}}-\mathrm{at}\;J(t)\geq J_{\mathrm{th}}$		Threshold model Ochando-Pulido et al. (2015)	[6]	-	Diffusive-Convective	Cross-flow	-	192	[59,189]
(5.3)	$J=\frac{e^{s}}{{1-e}^{-\mathrm{tp}*}}\sqrt{{\pi S}^{*}}\left[\mathrm{erf}\left(\sqrt{S^{*}{+t}_{p}*}\right)-\mathrm{erf}\left(\sqrt{S^{*}}\right)\right]$ Model parameters: S*, t_p_		Surface renovation theory Hasan et al. (2013)	[190]	Fermentation broths	Diffusive-Convective	Cross-flow	Unstirred cell	16	[120]
(5.4)	$\ln\left({J-J}_{\infty }\right){=\ln k}_{f}{+b}_{f}t$ ${J=J}_{\mathrm{ss}}k_{f}e^{{-b}_{f}t}$		Yee et al. (2009)	[191]	PEG	Diffusive-Convective	Cross-flow	Tubular	30	[171]
(5.5)	$J\left(f\right){=J}_{o}\exp\left\{\frac{-t}{f(t)}\right\}$ $f\left(t\right){=A}_{1}{+A}_{2}t$ Model parameter: A_1_, A_2_		Empirical model Mallubhotla and Belfort (1996)	[59]	Yeast	-	Dead-end	Unstirred cell	29	[68]
(5.6)	$J\left(f\right){=J}_{o}\exp\left\{\frac{-t}{f(t)}\right\}$ $f\left(t\right){=B}_{1}{+B}_{2}{t+B}_{3}t^{2}$	Modified Mallubhotla and Belfort	Modification empirical model Soler-Cabezas et al. (2015)	[68]	Waster water	-	Cross-flow	Hollow fiber	11	-
	$J\left(t\right){=C}_{1}+\frac{C_{2}}{\tan\left(C_{3}{t+C}_{4}\right)}$	Inverse Tangential								
	$J\left(t\right){=J}_{\mathrm{pss}}{+(J}_{o}{-J}_{\mathrm{pss}}{)e}^{-\left(D_{1}{t+D}_{2}t^{2}\right)}$	Exponential quadratic								
	$J\left(t\right){=E}_{1}+\frac{E_{2}}{\ln\left(E_{3}{t+E}_{4}\right)}$	Inverse logarithmic								
	$J\left(t\right){=F}_{1}\frac{\left(F_{2}{+e}^{F_{3}t}\right)}{\left(F_{4}{+e}^{F_{5}t}\right)}$	Exponential double								
	Model parameters: B, C, D, E, F									
(5.7)	Computational model of system dynamics (SD)		Zhu et al. (2016)	[8]	Raw water	-	Cross-flow	Stirred cell	0	-
(5.8)	Adaptive neuro-diffusive inference system model (ANFIS)		Salahi et al. (2015)	[7]	Wastewater	-	Cross-flow	Hollow fiber	-	-
(5.9)	PCA model of simultaneous multilevel analysis of components with invariant patterns (MSCA-P)		Modeling for Data Mining Klimkiewicz et al. (2016)	[15]	Enzymes	-	-	-	1	-
(5.10)	Neural network (ANN’s) per layer		Corbatón-Báguena et al. (2016)	[72]	PEG	-	Cross-flow	Tubular	6	-
(5.11)	Neural network (ANN’s) per layer		Díaz et al. (2017)	[12]	Water	-	Cross-flow	Tubular	0	-
(5.12)	$Y_{t}=\phi_{1}Y_{t-1}+\phi_{2}Y_{t-2}+\ldots +\phi_{p}Y_{t-p}{+\varepsilon }_{t}$	AR	ARIMA Ruby-Figueroa et al. (2017)	[69]	Fruit juices	-	Cross-flow	Tubular Hollow fiber	6	-
	$\Delta Y_{t}{=Y}_{t}{-Y}_{t-1}$	I								
	$Y_{t}{=\varepsilon }_{t}{+\theta }_{1}\varepsilon_{t-1}{+\theta }_{2}\varepsilon_{t-2}{+\ldots +\theta }_{q}\varepsilon_{t-q}$	MA								

BSA: Bovine serum albumin; PEG: Polyethylene glycol.

**Table 6:** Description of the UF membrane, operating conditions, and physicochemical characteristics of the fruit juices analyzed in this work *.

	Bergamot	Kiwi Fruit	Pomegranate	Reference
	DCQ II-006C	Koch Series-Cor TM HFM 251	FUC 1582	
Membrane characteristics and operation				
Membrane material	Polysulfone (PS)	Polyvinylidene fluoride (PVDF)	Triacetate cellulose (CTA)	-
Configuration	Hollow Fiber	Tubular	Hollow Fiber	-
Area (m^2^)	0.16	0.23	0.26	-
MWCO (kDa)	100	100	150	-
ΔP (bar)	1	0.85	0.6	-
Temperature (°C)	20	25	25	-
Flow (Lh^−1^)	114	800	400	-
Porosity (dimensionless)	0.0057	1.1	0.0007	
Tortuosity (dimensionless)	3	3	0.03	-
Membrane thickness (m)	4.7 × 10^−7^	2.0 × 10^−6^	0.00023	[34]
Pore density, N (number of pores m^−1^)	6.0 × 10^12^	4.0 × 10^16^	1.0 × 10^13^	[46]
Module length, L (mm)	330	406	136	[61]
Module diameter (m)	0.0021	0.025	0.0008	[30,46,192]
Hydraulic resistance (m^−1^)	3.6 × 10^12^	1.6 × 10^12^	2.1 × 10^12^	-
Hydraulic permeability (mPa^−1^s^−1^)	2.7 × 10^−10^	5.9 × 10^−10^	4.6 × 10^−10^	-
Fruit juices characteristics				
Total soluble solids (°Brix)	9.4	12.6	18.7	[30,38,43,193]
Titratable Acidity	53.86 (gL^−1^)	-	1.04 (% citric acid)	[30,38,43,193]
pH	2.40	3.19	3.61	[30,38,43,193]
Total phenolic compounds	660 (mg/L)	421.6 (mg/L)	1930 (mg GAE/100 L)	[30,38,43,193]
Turbidity (%)	33.67	-		[30,38,43,193]
Feed density, ρ (kgm^−3^)	1091	1070	1131	[194,195]
Feed viscosity, μ (Pa s)	0.0019	0.0014	0.0017	[31,196]
Concentration in food (%)	12	10.08	4.9	[27,33,36]

(*) The Supplementary Material includes the equations of density and viscosity as function of the concentration, used for the batch concentration analysis.

**Table 7:** Results of the simulation for selected models of bergamot juice clarification in terms of RMSE, MAPE, R^2^, and Shapiro–Wilk (S-W) and Kruskal–Wallis (K-W) residual analysis tests. Statistically validated models are in bold.

Models		RMSE	MAPE	R^2^	S-W	K-W
Concentration polarization model	**Davis (1992)/Shear-Induced Diffusion**	**0.80**	**11.76**	**91.08**	**0.00**	**0.10365**
Osmotic pressure models	**Keden & Katchalsky (1958)**	**0.25**	**5.70**	**99.17**	**0.0117**	**0.05**
	**Wijmans** **et al. (1984)**	**0.49**	**11.70**	**99.22**	**0.6855**	**0.0004**
Resistance in series models	**Hagen-Poiseuille (1839)**	**0.22**	**3.99**	**99.78**	**0.00034**	**0.8364**
	**De et al. (1997)**	**0.36**	**4.81**	**97.47**	**0.00**	**0.8692**
Fouling and adsorption models	Ho and Zydney (2000)	1.64	31.52	90.25	1.554 × 10^−15^	0.00
	Song (1998)/Dynamic model	1.51	35.90	97.56	0.00	0.00
	Mondal et al (2009)	1.76	18.23	87.01	0.0	0.00002
Non-Phenomenological models	**Yee et al. (2009)**	**2.03**	**28.91**	**84.91**	**0.000088**	**0.1038**
	**Ruby-Figueroa et al. (2017)/ARIMA models**	**0.40**	**8.24**	**97.92**	**2.99 × 10^−15^**	**0.056**

**Table 8:** Results of the simulation for selected models of kiwi juice clarification in terms of RMSE, MAPE, R^2^, and residual analysis tests Shapiro–Wilk (S-W) and Kruskal–Wallis (K-W). Statistically validated models are in bold.

Models		RMSE	MAPE	R^2^	S-W	K-W
Concentration polarization models	Davis (1992)/Shear-Induced Diffusion	2.91	22.35	52.86	0.00	1.213 × 10^−10^
Osmotic pressure models	Keden and Katchalsky (1958)	9.51	115.03	97.76	0.002	0.00
	**Wijmans** **et al. (1984)**	**0.33**	**3.14**	**97.98**	**0.075**	**0.45**
Resistance in series models	Hagen-Poiseuille (1839)	0.64	8.21	98.45	0.00	0.0032
	**De et al. (1997)**	**0.48**	**5.46**	**97.43**	**0.0012**	**0.2238**
Fouling and adsorption models	**Ho and Zydney (2000)**	**1.07**	**8.92**	**95.95**	**4.152 × 10^−12^**	**0.1015**
	Song (1998)/Dynamic model	3.94	43.51	67.94	0.00	0.00
	**Mondal et al (2009)**	**0.96**	**11.17**	**93.18**	**0.0**	**0.058**
Non-Phenomenological models	**Yee et al. (2009)**	**0.64**	**7.16**	**97.67**	**1.438 × 10^−13^**	**0.2047**
	**Ruby-Figueroa et al. (2017)/ARIMA models**	**0.33**	**3.74**	**98.98**	**0.0250**	**0.3801**

**Table 9:** Results of the simulation for selected models of pomegranate juice clarification in terms of RMSE, MAPE, R^2^, and residual analysis tests Shapiro–Wilk (S-W) and Kruskal–Wallis (K-W). Statistically validated models are in bold.

Models		RMSE	MAPE	R^2^	S-W	K-W
Concentration polarization models	**Davis (1992)/Shear-Induced Diffusion**	**1.64**	**27.56**	**85.58**	**2.** **22 × 10^−^^9^**	**0.8234**
Osmotic pressure models	Keden and Katchalsky (1958)	4.89	67.03	98.92	0.0001	3.581 × 10^−9^
	**Wijmans** **et al. (1984)**	**0.49**	**7.85**	**98.91**	**0.00**	**0.964**
Resistance in series models	**Hagen-Poiseuille (1839)**	**0.81**	**21.00**	**98.28**	**0.00**	**0.1974**
	**De et al. (1997)**	**0.72**	**16.64**	**96.73**	**2.33** **× 10** **^−13^**	**0.37255**
Fouling and adsorption models	**Ho & Zydney (2000)**	**2.01**	**51.69**	**75.91**	**2.93** **× 10** **^−12^**	**0.088**
	Song (1998)/Dynamic model	3.41	50.78	80.64	1.154 × 10^−14^	0.00
	**Mondal et al (2009)**	**1.60**	**17.45**	**92.40**	**0.0**	**0.3804**
Non-Phenomenological models	**Yee et al. (2009)**	**0.46**	**11.09**	**99.20**	**2.991** **× 10** **^−12^**	**0.2262**
	**Ruby-Figueroa et al. (2017)/ARIMA models**	**0.25**	**4.08**	**99.70**	**0.00**	**0.6320**

## Data Availability

The data presented in this study are available on request from the corresponding authors (Elizabeth Troncoso, René Ruby-Figueroa).

## Supplementary Materials

The following are available online at https://www.mdpi.com/article/10.3390/membranes11050368/s1, Table S1: Description of equations used for performance analysis of selected models to predict permeate flux in ultrafiltration processes.

## Nomenclature

A	Membrane area: m^2^	Sh	Sherwood number, dimensionless
A_d_	Membrane area in cell, m^2^	Sc	Schmidt number, dimensionless
C_b_	Bulk concentration, kgm^−3^	Pe	Peclet number, dimensionless
C_CL_	Boundary layer concentration, kgm^−3^	W_d_	Stability factor with respect to deposition (parameter in No. 4.4, Table 4)
C_g_	Gel concentration, kgm^−3^	s	Sedimentation coefficient
C_gv_	Concentration gel layer in volume, m^3^	t	Time, s
C_m_	Intrinsic concentration, kgm^−3^	t_R_	Fouling phase time, s
C_p_	Permeated concentration, kgm^−3^	τ	Tortuosity, dimensionless
C_0v_	Feed concentration, kgm^−3^	T	Temperature, °C
D_m_	Equivalent diffusion diameter of macromolecules (parameter in No. 1.11), m	U	Flow velocity, ms^−1^
D	Diffusivity, m^2^s−^1^	U_0_	Initial flow velocity, ms^−1^
d	Diameter of the module, m	v_(L)_	Average permeate velocity on the length of the filter channel, ms^−1^
d_h_	Hydraulic diameter, m	v_f_	Local filtrate velocity (parameter No. 1.8), ms^−1^
d_p_	Pore diameter, m	X(t)	Position change of the equilibrium zone (parameter in No. 4.5)
F	Intermolecular interactions	b	Radius of the stirred cell (parameter of No. 1.8)
f_c_	Marchetti correction factor	V	Permeate volume (parameter in No. 3.10), L
f_cp_	Capilar effect	V_c_	Permeate volume at the reference time point (parameter in No. 3.10), L
b	Inverse of the solute density (parameter of No. 1.9)	V	Permeate volume, m^3^
H	Height of liquid over membrane (parameter of No. 1.9), m	V_0_	Initial permeate volume, m^3^
η	Non-dimensional distance (parameter of No. 1.9) = x/H	v	Specific partial volume, kgm^3^
ϕ	Non-dimensional concentration (parameter of No. 1.9) = c/co	v_0_	Specific partial initial volume, kgm^3^
f_d_	Dipole effect	VRF	Volume reduction factor, dimensionless
f_e_	Steric effect	v	Kinematic viscosity, m^2^s^−1^
H	Thickness of the gel layer, m	x_i_	Proportional parameter of permeability in No. 1.3
J	Permeate flux, ms^−1^	∆x	Membrane thickness, m
J_f_	Final permeate flux, ms^−1^	X_12_	Flory-Huggins parameter
J_lim_	Limit permeate flux, ms^−1^	z*	Axial position for osmotic pressure, m
J_∞_	Saturation (equilibrium) volumetric permeate flux, m^3^m^−2^s^−1^		
J_ss_	Steady-state permeate flux, ms^−1^	Greek symbols	
J_W_	Flux of pure water permeate, ms^−1^	δ	Thickness of the boundary layer, m
J*	Hydraulic lifting speed, ms^−1^	∆π	Osmotic pressure, Pa
J^o^	Balance between solute input and output,	γ	Shear rate, ms^−1^
k	Mass transfer coefficient, ms^−1^	γm	Shear rate at the wall (parameter in No. 1.11), s^−1^
k_o_	Ideal mass transfer coefficient, ms^−1^	Ɛ	Porosity of the membrane, dimensionless
K	Boltzmann constant (parameter in No. 1.11), Jmol^−1^K^−1^	α	Specific resistance of the deposit on membrane, kgm^2^
L	Length of the module, m	ϵ	Specific gel resistance, m^−2^
L_p_	Membrane permeability, mPa^−1^s^−1^	Ɛ_CL_	Boundary layer porosity, dimensionless
L_ph_	Effective permeability reverse flow,	β	Parameter of No. 1.5
m_p_	Deposited cake weight, kg	σ	Reflection coefficient
MW	Membrane cut-off limit, gmol^−1^	Ɛ_g_	Solidity of the gel layer, %
∆P	Transmembrane pressure, Pa	ε_st_	Steady-state value of the average solidity, %
Q	Flow rate, m^3^s^−1^	μ_0_	Initial viscosity, Pa s
R_m_	Membrane resistance, m^−1^	μ_b_	Viscosity in the bulk, Pa s
R_M_	Fouled membrane resistance, m^−1^	μ	Viscosity, Pa s
R_ad,ss_	Adsorption resistance, m^−1^	ρ	Feed density, kgm^−3^
R_cp_	Concentration polarization resistance, m^−1^	ρ_pol_	Membrane polymer density, kgm^−3^
R_cp,ss_	Steady-state concentration polarization resistance, m^−1^	γ_a_	Axial speed, ms^−1^
Re	Reynolds number, dimensionless	υ_p_^o^	Osmotic pressure limiting flux (m^3^ m^−2^ s^−1^)
R_f_	Irreversible resistance, m^−1^	X_12_	Flory–Huggins interaction parameter
R_g_	Gel resistance, m^−1^	ϕ	Volume fraction of particles at the distance x from the membrane surface
R_m_	Hydraulic resistance, m^−1^	ϕ	1/Jlim (parameter of No. 3.11), m^2^sm^−3^
$R_{m}^{*}$	Experimental resistance (constant ΔP), m^−1^		
R_m i−1_	Accumulated resistance at t_i−1_,		
$R_{\mathrm{ps}}^{*}$	Concentration polarization differential resistance, dimensionless		
R_t_	Total resistance, m^−1^		
r_CL_	Specific resistance of the boundary layer, m^−1^		
r_i_	Cell radius, m		
r_o_	Initial cell radius, m		
r_p_	Particle radius, m		
r_pp_	Membrane pore radius, m		
