# Supplementary material for: Prediction of Permeate Flux in Ultrafiltration Processes: A Review of Modeling Approaches
Source: Membranes (Basel). 2021 May 18;11(5):368. doi: 10.3390/membranes11050368 (PMC8158366; doi:10.3390/membranes11050368)
Supplement: Supplementary file 1 [file membranes-11-00368-s001.zip › membranes-1156760-supplementary.pdf]

SUPPLEMENTARY MATERIAL

Table 1: Description of equations used for performance analysis of selected models to predict permeate flux in ultrafiltration processes

Equation	Description	Nomenclature	Models tested with this equation	Reference
$\mu = 8{,}8E\text{-}4 + 1{,}2E\text{-}5\ Cb + 1{,}41E\text{-}7\ Cb^2 + 8{,}7E\text{-}10\ Cb^3$	Equation used for viscosity estimation	μ = Viscosity<br>Cb = Bulk concentration	- Shear-Induced (Davis)<br>- Dynamic (Song)<br>- Kenden & Katchalsky<br>- Wijmans *et al*<br>- Hagen-Poiseuille<br>- De & Bhattacharya<br>- Ho & Zydney	[1]
$\frac{Cb}{Cbo} = \frac{Vo}{Vo\text{-}V}$	Equation used for estimations of feed concentration and permeate volume	Cb = Bulk concentration<br>Cbo= Feed concentration<br>V= Total permeate volume<br>Vo= Initial volume	- Shear-Induced (Davis)<br>- Dynamic (Song)<br>- Hagen-Poiseuille<br>- De & Bhattacharya	[2]
$D = \sum D_i\ Z_i$	Equation used for diffusivity estimation	D = Diffusivity<br>D_i = Diffusivity of solid i<br>Z_i = Weight fraction of suspended solid i<br>i = Refers to each existing solid in the juice	- Shear-Induced (Davis)<br>- Wijmans *et al*<br>- De & Bhattacharya	[1]
$\rho = 999{,}7 + 5{,}1E\text{-}1\ Cbo + 2{,}1E\text{-}3\ Cbo^2$	Equation used for density estimation	ρ = Density<br>Cbo= Feed concentration	- Shear-Induced (Davis)<br>- Dynamic (Song)<br>- Kenden & Katchalsky<br>- Wijmans *et al*<br>- Hagen-Poiseuille<br>- De & Bhattacharya<br>- Ho & Zydney<br>- Mondal & De<br>- Yee *et al*	[1]
$\varepsilon = N \frac{\pi}{4} d_p^2$	Equation used for porosity estimation	ε = Porosity<br>N = Pore density<br>dp = Pore diameter	- Wijmans *et al*<br>- Hagen-Poiseuille<br>- De & Bhattacharya	[3]
$J = \frac{\lvert\Delta P\rvert - \lvert\Delta\pi\rvert}{\mu R_m}$	Equation used to determine osmotic pressure (Δπ)	J = Flux of permeate<br>ΔP = Transmembrane pressure<br>μ = Viscosity<br>R_m = Hydraulic resistance	- Kenden & Katchalsky<br>- Wijmans *et al*	[4]

$Sh = \frac{k\,d}{D}$	Sherwood number used for estimation of the mass transfer coefficient	Sh = Sherwood number<br>k = Mass transfer coefficient<br>d = Diameter module<br>D = Diffusivity	- Wijmans *et al*<br>- De & Bhattacharya	[5]
$r_p = 180\,\frac{(1-\varepsilon)^2}{a_p^2 \varepsilon 3}$	Carman-Kozeny' equation used to determine the specific resistance (r_p)	ε = Porosity of the concentration polarisation layer<br>a_p= Average solute particle diameter	Wijmans *et al*	[6]
$R_p = r_p\ \delta_p$	Resistance of concentration polarisation layer (R_p)	r_p = Specific resistance<br>δ_p = Thickness of concentration layer	- Wijmans *et al*<br>- Ho & Zydney	[6]
$\delta_p = \frac{k}{D}$	Thickness of concentration layer (δ_p)	k = Mass transfer coefficient<br>D = Diffusivity	- Wijmans *et al*<br>- De & Bhattacharya	[3]
$\delta_* = \left(\frac{144\,D\,L\,C_b^*}{\left(3+(\frac{1}{n})\right) U_o d^2 C_g^*} \right)^{1/3} x_*^{1/3}$	Equation used for estimation of the non-dimensional thickness of the concentration boundary layer (δ_*)	D = Effective diffusivity of the gel forming solutes<br>L = Module lenght<br>Cb* = Non-dimensional bulk concentration<br>n = Flow behavior index<br>Uo = Maximum fluid velocity (centerline velocity)<br>d = Module diameter<br>Cg* = Non-dimensional gel layer concentration<br>X* = x/L (x=distance along x-axis)	De & Bhattacharya	[7]
$Sh = 1{,}145 \left(3+\frac{1}{n}\right)^{1/3} \left(Re{*}Sc{*}\frac{d}{L}\right)^{1/3} \left(e^{(-2/3)\alpha C_0 \left(C_g^*-C_b^*\right)}\right)^{0.1}$	Sherwood number – Hollow fiber configuration	Re= Reynolds number<br>Sc = Schmidt number<br>Cb* = Non-dimensional bulk concentration<br>Cg* = Non-dimensional gel layer concentration<br>L = Module lenght<br>d = Module diameter<br>n = Flow behavior index	De & Bhattacharya	[7]
$Sh = 1{,}816 \left(Re{*}Sc{*}\frac{d}{L}\right)^{1/3} \left(e^{(-2/3)\alpha C_0 \left(C_g^*-C_b^*\right)}\right)^{0.14} \left(\frac{C_g^*}{C_b^*}\right)^{1/3}$	Sherwood number – Tubular configuration	Re= Reynolds number<br>Sc = Schmidt number<br>Cb* = Non-dimensional bulk concentration	De & Bhattacharya	[8]

		Cg^* = Non-dimensional gel layer concentration<br>L = Module lenght<br>d = Module diameter<br>n = Flow behavior index		
$Rg = \beta(1-\varepsilon_g)\rho_g H$	Gel layer resistance (Rg)	ρ_g = Gel layer density<br>H = Gel layer height<br>ε_g= Gel layer porosity<br>β = Specific gel resistance	De & Bhattacharya	[7]
$R^*_{CPB} = \exp(k_1 t) - 1$	Equation used for estimation of the non-dimensional complete pore blocking resistance (R^*_{CPB})	k_1= Complete pore blocking constant<br>t = Time	Mondal & De	[9]
$R^*_c = \frac{R_p}{R_m}$	Equation used for estimation of the non-dimensional cake resistance (R^*_c)	Rp = Resistance of concentration polarisation layer<br>Rm= Hydraulic resistance of membrane	Mondal & De	[9]

[1] P.K. Bhattacharya, S. Agarwal, S. De, U.V.S. Rama Gopal, Ultrafiltration of sugar cane juice for recovery of sugar: Analysis of flux and retention, Sep. Purif. Technol. 21 (2001) 247–259. https://doi.org/10.1016/S1383-5866(00)00209-4.

[2] T. Furukawa, K. Kokubo, K. Nakamura, K. Matsumoto, Modeling of the permeate flux decline during MF and UF cross-flow filtration of soy sauce lees, J. Memb. Sci. 322 (2008) 491–502. https://doi.org/10.1016/j.memsci.2008.05.068.

[3] M. Cheryan, Ultrafiltration and Microfiltration Handbook, 1998. https://doi.org/97-62251.

[4] J. Huter, Model-Based Design and Process Optimization of Continuous Single Pass Tangential Flow Filtration Focusing on Continuous Bioprocessing, Processes. (2019).

[5] S. Mondal, Chhaya, S. De, Modeling of cross flow ultrafiltration of stevia extract in a rectangular cell, J. Food Eng. 112 (2012) 326–337. https://doi.org/10.1016/j.jfoodeng.2012.05.002.

[6] K. Damak, A. Ayadi, P. Schmitz, B. Zeghmati, Modeling of crossflow membrane separation processes under laminar flow conditions in tubular membrane, Desalination. 168 (2004) 231–239. https://doi.org/10.1016/j.desal.2004.07.003.

[7] S. Mondal, A. Cassano, C. Conidi, S. De, Modeling of gel layer transport during ultrafiltration of fruit juice with non-Newtonian fluid rheology, Food Bioprod. Process. 100 (2016) 72–84. https://doi.org/10.1016/j.fbp.2016.06.012.

[8] S. Mondal, A. Cassano, F. Tasselli, S. De, A generalized model for clarification of fruit juice during ultrafiltration under total recycle and batch mode, J. Memb. Sci. 366 (2011) 295–303. https://doi.org/10.1016/j.memsci.2010.10.015.

[9] S. Mondal, S. De, Generalized criteria for identification of fouling mechanism under steady state membrane filtration, J. Memb. Sci. (2009). https://doi.org/10.1016/j.memsci.2009.08.015.
